# Cost-effectiveness of interventions for the prevention and control of COVID-19: Systematic review of 85 modelling studies

**DOI:** 10.7189/jogh.12.05022

**Published:** 2022-06-15

**Authors:** Lihui Zhou, Wenxin Yan, Shu Li, Hongxi Yang, Xinyu Zhang, Wenli Lu, Jue Liu, Yaogang Wang

**Affiliations:** 1 School of Public Health, Tianjin Medical University, Tianjin, China; 2 Department of Epidemiology and Biostatistics, School of Public Health, Peking University, Beijing, China; 3 School of Management, Tianjin University of Traditional Chinese Medicine, Tianjin, China; 4 School of Basic Medical Sciences, Tianjin Medical University, Tianjin, China; 5 Institute for Global Health and Development, Peking University, Beijing, China; 6 Health Science and Engineering College, Tianjin University of Traditional Chinese Medicine, Tianjin, China

## Abstract

**Background:**

We aimed to quantitatively summarise the health economic evaluation evidence of prevention and control programs addressing COVID-19 globally.

**Methods:**

We did a systematic review and meta-analysis to assess the economic and health benefit of interventions for COVID-19. We searched PubMed, Embase, Web of Science, and Cochrane Library of economic evaluation from December 31, 2019, to March 22, 2022, to identify relevant literature. Meta-analyses were done using random-effects models to estimate pooled incremental net benefit (INB). Heterogeneity was assessed using *I^2^* statistics and publication bias was assessed by Egger’s test. This study is registered with PROSPERO, CRD42021267475.

**Results:**

Of 16 860 studies identified, 85 articles were included in the systematic review, and 25 articles (10 studies about non-pharmacological interventions (NPIs), five studies about vaccinations and 10 studies about treatments) were included in the meta-analysis. The pooled INB of NPIs, vaccinations, and treatments were $1378.10 (95% CI = $1079.62, $1676.59), $254.80 (95% CI = $169.84, $339.77) and $4115.11 (95% CI = $1631.09, $6599.14), respectively. Sensitivity analyses showed similar findings.

**Conclusions:**

NPIs, vaccinations, and treatments are all cost-effective in combating the COVID-19 pandemic. However, evidence was mostly from high-income and middle-income countries. Further studies from lower-income countries are needed.

The COVID-19 caused by severe acute respiratory syndrome coronavirus 2 (SARS-CoV-2) infection has wreaked a pandemic all over the world [1], leaving a trail of ruined economies and public health disasters in its wake [2]. The disease burden of COVID-19 and the costs of curbing COVID-19 are of great concern. As of April 12, 2022, there have been 497 million confirmed cases of COVID-19 and 6 million deaths worldwide, according to the World Health Organisation [3]. It was estimated that a single symptomatic SARS-CoV-2 infection would have a median direct medical cost of US$3045 during the infection, which would increase to $14 366 in case of hospitalization [4].

Comprehensive interventions have been taken to prevent and control COVID-19. Among them, non-pharmacological interventions (NPIs), including travel restrictions, masks, social distancing, and public education on preventive measures and school closures have been used worldwide to suppress the COVID-19 pandemic [5]. Although vaccine coverage has increased rapidly, many regions remain at risk of COVID-19-related health pressures because of inequitable access to COVID-19 vaccines globally [6]. Medication use for treatment and prevention is still an important strategy alongside vaccine rollout [7]. Remdesivir and other antivirals are increasingly prescribed in hospital settings. Given the high cost of the drugs, optimal timing of treatment is critical to improve outcomes and maintain cost-effectiveness [8]. Studies have shown that these strategies could effectively reduce infections and control transmission [9-11], but they might also increase the burden of medical expenditure and lead to significant reductions in baseline productivity [12].

The crisis requires significant government support and effort to recover all aspects of the economy [13]. It is necessary to assess how these interventions perform when cost-effectiveness is considered [12]. However, there are discrepancies between current evidence on the cost-effectiveness of COVID-19 interventions. Studies in South African health care settings show that NPIs such as household contact tracing, isolation, mass symptom screening, and quarantining household contacts who test negative reduced mortality by 94% and were cost-effective with the ICER lower than the threshold of US$3250 per year of life saved [14]. However, a continuing lockdown was not cost-effective in the UK, as it would lead to a quality-adjusted life-year (QALY) value of 7 to 125 times higher than recommended by the National Institute for Health and Care Excellence guidelines [15]. Meta-analyses of drug treatments or vaccines tended to focus on effectiveness and safety, rather than economic benefit analysis [16-18]. One systematic review of economic evaluation of programs against COVID-19 does exist, however, but was done at a time when vaccines were not yet available [19,20].

In this systematic review and meta-analysis, we aimed to summarise the health economic evaluation evidence of prevention and control programs addressing COVID-19 globally and experimentally demonstrate the pooled individual-level incremental net benefits (INB) of interventions in different subgroups with varying characteristics. The results of this analysis could provide insight for policymakers in optimizing scarce health care and public property resources in recent public health interventions and help them prepare for subsequent waves of COVID-19 or other pandemics to come.

## METHODS

### Search strategy and selection criteria

This study followed the Preferred Reporting Items for Systematic Reviews and Meta-Analyses (PRISMA-P) guideline [21]. The review protocol was registered at PROSPERO (ID CRD42021267475).

Four electronic databases, including PubMed, Web of Science, Embase and Cochrane Library, were searched for publications on the cost-effectiveness analysis of control and treatment interventions against COVID-19, from December 31, 2019, to March 22, 2022. The objective of our systemic review was to summarize and experimentally quantify the economic and clinical benefits of control and treatment interventions for COVID-19. We constructed search terms based on the population, intervention, comparison, outcome, and study design (PICOS) framework (ie, COVID-19, incremental cost-effectiveness ratio, economic evaluation, and decision analysis). We used medical subject heading (MeSH) terms and keywords for “cost” and “COVID-19”. The specific search strategies can be found in Appendix S1 in the **Online Supplementary Document**. The inclusion criteria of this study were 1) reporting the cost-effectiveness of control or treatment strategies against COVID-19 using either cost-effectiveness analysis (CEA), cost-utility analysis (CUA) or cost-benefit analysis (CBA); 2) no language or geographic restrictions on search strategies were set; 3) results reporting one of the index of economic evaluation analysis, including incremental cost-effectiveness ratio (ICER), net benefit, incremental net benefit, benefit-cost ratio, etc. The exclusion criteria were: 1) merely disease burden studies; 2) comments, letters, replies or conference abstracts.

### Data collection, preparation, and quality assessment

Duplicate articles were removed. Titles and abstracts screening, full-text review, and data extraction were done by two reviewers (ZLH and YWX) independently based on inclusion and exclusion criteria. Discrepancies between the two reviewers were resolved by a third reviewer (LJ) and the final decision was made by consensus.

Basic study characteristics were collected, including the author, published year, country, population characteristics and size, studied and compared interventions, time horizon, type of economic evaluation (CEA, CUA, or CBA), perspective, results of base-case outcomes, and sensitivity analysis. The primary pooled parameter in this study was individual-level INB, define as INB = K × ΔE-ΔC, where K is the willingness-to-pay threshold and ΔE and ΔC were the incremental (difference in cost between intervention and comparator) cost and effect, respectively [22]. A positive INB showed favouring intervention (ie, intervention is cost-effective), whereas a negative INB indicated favouring the comparator (ie, intervention is not cost-effective). The higher INB indicated that greater benefit would be obtained if the interventions were implemented [23].

We extracted data needed for estimating the INB of each intervention, the mean, and dispersions (ie, standard deviations (SDs), standard errors (SEs), and 95% confidential intervals (CIs)) of cost, incremental cost, effectiveness, incremental effectiveness, ICER, and willingness-to-pay threshold. In some cases, the related data were extracted from probabilistic sensitivity analyses, like the cost-effect plane with scatterplot graphs. For studies not reported directly, we calculated the INB and its 95% CI based on the 5 scenarios in line with previous recommendations using the data extracted from the articles (Appendix S2 in the **Online Supplementary Document**) [22,24].

To standardize pooling parameters, all INBs were converted to US dollars using the exchange rate mentioned in the original articles, or the exchange rate of the model setting year. A discount rate was not applied since the publication interval was short (2020 to 2022). Further, the estimated INB was divided by the simulated sample size from the original articles to eliminate the influence caused by different sizes of modelling cohorts.

To assess the quality and risk of bias of the studies, the Consolidated Health Economic Evaluation Reporting Standards (CHEERS) checklist was used [25]. The assessment was done based on the following criteria: study perspective, comparator description, time horizon, description of discounting of cost and outcome, description of model with figures provided, clear reporting of study population, reporting ICER and its unit, sensitivity analysis, and disclosure of funding sources and any conflict of interest. The quality of studies was ranked by the scores of CHEERS, with quality categorized as excellent (≥85%), very good (70% ~ 85%), good (55% ~ 70%), and poor (<55%).

### Statistical analyses

The INB along with its variance were estimated and pooled across studies by type of control and treatment (ie, NPIs, COVID-19 vaccines and their strategies, COVID-19 treatment). According to the central limit theorem, the incremental net benefit (INB) is asymptotically normal [26] and the pooled INB is also asymptotically normal when the mean and variance are known. Based on these assumptions, the total INB was estimated using an inverse variance method if heterogeneity was not present; otherwise, a random-effect model with the standard DerSimonian and Laird method was applied [27]. The heterogeneity of the INB across studies was assessed by *I^2^* statistics. The heterogeneity was considered as present if *I^2^* was greater than 50%.

We analysed data in different subgroups of types of intervention and comparator, categories of income of economy, perspective, and time horizon. Publication bias was assessed using a funnel plot and Egger’s test. The trim-and-fill algorithm was used to estimate the number of studies potentially missing from the meta-analysis when publication bias was detected.

For the sensitivity analysis, we performed influence analysis by omitting one study. Orwin's fail-safe N test was used to test whether the non-pooled lectures would change the main results. Data pooling was analysed by STATA version 13.0 (Stata Corp LP, College Station, TX, USA) and R software version 4.1.0 (R Foundation for Statistical Computing, Vienna, Austria), with package “metafor”. A two-sided *P-*value of <0.05 was considered statistically significant.

## RESULTS

**Figure 1:**
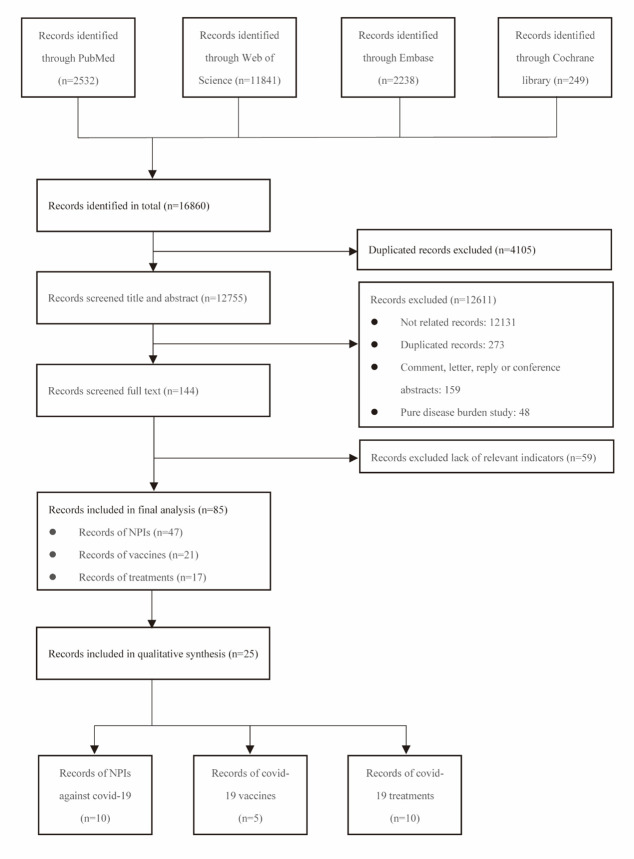
Details of study selection for meta-analysis NPIs – non-pharmacological interventions.

A total of 16 860 records were retrieved from the four databases by the search strategy mentioned above. 4105 duplicates were excluded. After screening the titles and abstracts, we excluded 12 611 comments, letters, replies, conference abstracts, pure disease burden studies and other topically irrelevant studies. 144 articles underwent a full-text review and 59 were excluded for lacking targeted data or meeting any one of the exclusion criteria (**Figure 1**).

Ultimately, 85 articles were included in this systematic review (Appendix S3 in the **Online Supplementary Document**). Most of these studies were from the US (n = 30), followed by the UK (n = 8), South Africa (n = 4), and China (n = 4). Most included studies were conducted from a health care system (n = 29) and societal perspective (n = 20) while a couple of studies were carried out from the third-party payer and public payer perspectives. 52 studies evaluated interventions against COVID-19 in the general population, 20 studies assessed strategies among patients with or suspected of having COVID-19, six studies focused on the health care workers (HCWs), and four studies were on the college students. Other studies included the homeless in shelters, older adults aged above 65, and people in sports competitions. 47 studies evaluated NPIs including universal PCR testing, contact tracing, isolation centres, screening, social distancing, masks, lockdowns, business closures, reinfection control, and media campaigns. 21 articles examined vaccine types, coverage rates, sensitivity rates, and other indicators. 17 studies focused on ICU allocation and usage of anti-inflammatory drugs including remdesivir, dexamethasone, and tocilizumab individually or in combination.

In total, 25 articles were eligible for meta-analysis. Among them, 10 articles assessed the cost-effectiveness of NPIs against COVID-19 [14,28-36]. 37 NPIs were included with varying epidemic scenarios. 10 articles were about the treatments of COVID-19 patients [37-46], and 18 treatments were pooled. five studies (seven interventions) examined the cost-effectiveness of COVID-19 vaccines or vaccine strategies [47-51].

The risk of bias assessment was performed using the CHEERS checklist (Appendix S4 in the **Online Supplementary Document**). The average quality score was 77% (maximum score = 96%, minimum score = 50%). 94.1% of studies were assessed as having at least good quality and 36.5% of studies had excellent quality.

### NPIs against COVID-19

**Figure 2:**
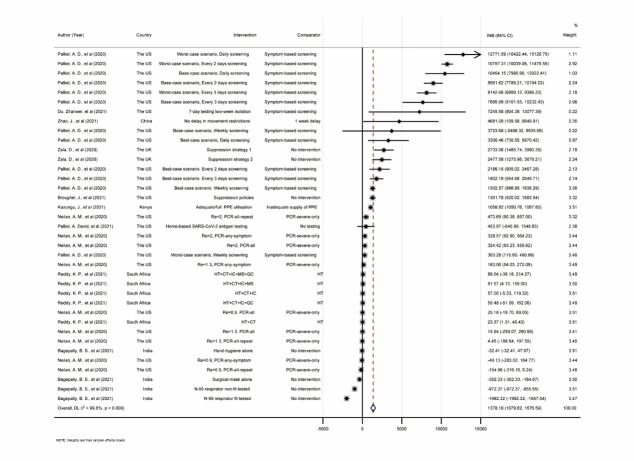
Forest plot of pooling the incremental net benefit of non-pharmacological interventions against COVID-19. INB – incremental net benefit, CI – confidential interval, Re – effective reproductive number, SARS-CoV-2 – severe acute respiratory syndrome coronavirus 2, PPE – personal protective equipment, HT – health care testing alone, CT – contact tracing, IC – Isolation centres, MS – mass symptom screening, QC – quarantine centres, PCR – polymerase chain reaction. 1) Suppression 1 and Suppression 2 involve the addition of more extensive controls to those implemented under mitigation, namely general social distancing and closure of schools and universities; Suppression 1: triggered “on” when there are 100 ICU cases in a week and “off” when weekly cases halve to 50 cases; Suppression 2, triggered “on” when there are 400 ICU cases in a week and “off” when weekly cases halve to 200 cases.

The INBs of NPIs were estimated for 10 individual studies (37 interventions), with large heterogeneity (*I^2^ =* 99.8%). They were then pooled using a random-effects model with a pooled INB (INBp) of US$1378.10 (95% CI = US$1079.62, US$1676.59), indicating that putting these interventions into practice would benefit the participants (**Figure 2**). In the forest plot, higher INBs were seen in PCR screening compared to symptom-based screening, with no delay in movement restrictions and suppression compared with no interventions.

The results of subgroup analyses showed that higher pooled INBs were seen in subgroups of screening and suppression interventions, symptom-based screening, high-income economies, and shorter time horizons (<365 days). Positive INBp were observed in most cases, except for the public payers’ perspective subgroup (**Table 1**). A funnel plot was constructed indicating funnel asymmetry (*P* < 0.001) (Appendix S5 in the **Online Supplementary Document**). According to the trim-and-fill method results, no missing interventions exist in the left or right of the funnel (Appendix S5 in the **Online Supplementary Document**). A sensitivity analysis was performed by omitting one intervention (Appendix S6, Figure S3 in the **Online Supplementary Document**). The pooled INBs were all above 0 and the lowest and highest INBp was observed when the interventions of Adequate PPE utilization [36] and the combined intervention of health care testing and contact tracing [14] were excluded. The fail-safe number was 37, indicating that 37 negative studies or interventions might change the current results. Among the remaining 38 un-pooled studies about NPIs against COVID-19, five reported the interventions were not cost-effective [15,52-55], 21 concluded that the interventions were cost-effective or cost-saving [56-76], 10 reported that parts of the studied interventions were cost-effective or cost-effective with minor benefit [77-85], and one study concluded the results were uncertain and conditional [86].

**Table 1:** Subgroup meta-analysis of NPIs against COVID-19*

Subgroups	No. of studies	No. of interventions	INBp and 95% CI (US$)	(%)	p (for )
**Type of interventions**					
PPE and others	2	5	-456.18 (-1400.38, 488.02)	100.0	<0.001
Multiple interventions	2	6	51.31 (8.91, 93.72)	44.8	0.107
Suppression	3	4	2156.00 (1114.40, 3197.59)	66.7	0.029
Screening	3	22	2390.89 (1932.92, 2848.85)	98.6	<0.001
**Type of comparators**					
No intervention	3	7	117.91 (-425.72, 661.55)	99.5	<0.001
Symptom-based screening	2	13	6009.59 (3865.77, 8153.40)	99.0	<0.001
PCR-severe-only	1	9	93.91 (-7.99, 195.81)	71.4	<0.001
No testing	1	1	453.97 (-640.89, 1548.83)	-	-
HT	1	5	33.02 (13.52, 52.51)	0.0	0.441
Inadequate supply of PPE	1	1	1058.82 (1050.29, 1067.36)	-	-
1 week delay in movement restrictions	1	1	4681.00 (-89.17, 9451.17)	-	-
**Income of economies**					
Low-middle income economies	2	5	-456.18 (-1400.38, 488.02)	100.0	<0.001
Upper-middle income economies	2	6	47.98 (11.39, 84.57)	32.4	0.193
High income economies	6	26	2381.71 (1950.64, 2812.79)	98.5	<0.001
**Perspectives**					
Public payers	1	4	-833.05 (-1470.73, -195.37)	99.7	<0.001
Health care sector	3	15	165.76 (-190.83, 522.35)	59.7	0.002
Societal	2	2	3151.07 (-3362.17, 9664.32)	78.1	<0.001
Not mentioned	4	16	5102.96 (3503.21, 6702.71)	98.8	<0.001
**Time horizon**					
≥360 d	3	10	-196.74 (-705.59, 312.11)	99.9	<0.001
<360 d	4	23	2416.85 (1959.52, 2874.17)	98.6	<0.001
Not mentioned	3	4	2156.00 (1114.40, 3197.59)	66.7	0.029
**Combined**	10	37	1378.10 (1079.62, 1676.59)	99.8	<0.001

NPI – non-pharmacological interventions, INBp – pooled incremental net benefit, PPE – personal protective equipment, CI – confidential interval, PCR – polymerase chain reaction, HT – health care testing alone,

*References in each subgroup were listed in forest plots of Appendix S7 in the **Online Supplementary Document**.

### Vaccinations against COVID-19

**Figure 3:**
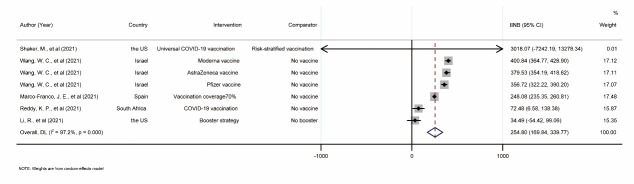
Forest plot of pooling the incremental net benefit of COVID-19 vaccination and vaccination strategies. INB – incremental net benefit, CI – confidential interval.

The INBs of vaccines and vaccination strategies were estimated for five individual studies with large heterogeneity (*I^2^ =* 97.2%). They were then pooled using a random-effects model with a pooled INB of US$254.80 (95% CI = US$169.84, US$339.77), indicating that putting these interventions into practice would benefit the participants (**Figure 3**).

The results of the subgroup analysis were consistent with the main results (Appendix S7: Table S3, Figure S15-S18 **in the** **Online Supplementary Document**). A funnel plot was constructed indicating symmetry of the funnel (*P* = 0.993) (Appendix S5 in the **Online Supplementary Document**), indicating that publication bias was less likely. The sensitivity analysis was performed by omitting one intervention (Appendix S6: Figure S4 in the **Online Supplementary Document**). The pooled INBs were all above 0, and the lowest and highest INBp was observed when Moderna vaccination [50] and booster vaccination [51] were excluded. The fail-safe number was seven, indicating seven studies or interventions with negative results could change the current results. Of the remaining 15 studies on COVID-19 vaccination, 12 concluded that COVID-19 vaccinations were cost-effective [87-98] and two suggested that parts of the vaccination strategies were optimal [99,100]. Only one study reported the conclusion depended on the primary outcome of interest [101].

### Treatments of COVID-19

**Figure 4:**
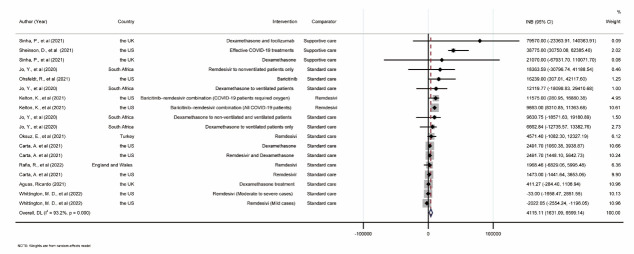
Forest plot of pooling the incremental net benefit treatments of COVID-19 patients. INB – incremental net benefit, CI – confidential interval.

The INBs of treatments were estimated for 10 individual studies with large heterogeneity (*I^2^ =* 93.2%). They were pooled using a random-effects model with a pooled INB of US$4115.11 (95% CI = US$1631.09, US$6599.14), indicating that putting these interventions into practice would benefit participants (**Figure 4**).

Furthermore, positive INBp was observed in most subgroup analyses, while greater INBp was seen in subgroups of supportive care comparator and lifetime horizon. (**Table 2**). A funnel plot was constructed indicating funnel asymmetry (*P* < 0.001) (Appendix S5 in the **Online Supplementary Document**). Six missing interventions on the left side of the funnel were imputed and the INBp was still above 0. In sensitivity analysis, the pooled INBs were all above 0. The lowest and highest INBp was observed when Baricitinib–remdesivir combination treatment compared to Remdesivir [42] and Dexamethasone treatment [37] was omitted. The fail-safe number was 18, indicating that 18 studies or interventions with negative results might change the current results. Of the remaining seven un-pooled studies about COVID-19 treatment, two concluded that COVID-19 vaccinations were cost-effective [11,102-105]. For lower-income economies like Kenya, investments in essential care before advanced critical care should be prioritised [106], and only one study reported general ward and intensive care was not cost-effective compared to general ward only [107].

**Table 2:** Subgroup meta-analysis of treatments of COVID-19*

Subgroups	No. of studies	No. of interventions	INBp and 95% CI (US$)	(%)	p (for )
**Type of comparators**					
Supportive care	2	3	39 676.66 (24 377.42, 54 975.89)	0.0	0.579
Standard care	7	13	1212.96 (-340.51, 2766.42)	80.3	<0.001
Remdesivir	1	2	9919.01 (8417.77, 11420.25)	0.0	0.691
**Income of economies**					
Upper-middle income economies	2	5	6080.27 (613.37, 11547.17)	0.0	0.905
High income economies	8	13	3757.93 (1100.95, 6414.92)	95.1	<0.001
**Perspectives**					
Health payer or health system	6	12	1553.76 (-267.05, 3374.58)	86.2	<0.001
Payor	3	4	9695.84 (8234.46, 11157.22)	0.0	0.413
Not mentioned	1	2	52 759.54 (-7492.61, 113 011.68)	0.0	0.343
**Time horizon**					
Lifetime	6	9	8082.70 (1938.61, 14226.78)	96.5	<0.001
Others (3 y or lower)	4	9	1770.27 (541.24, 2999.30)	39.8	0.102
**Combined**	10	18	4115.11 (1631.09, 6599.14)	93.2	<0.001

CI – confidential interval, INBp – pooled incremental net benefit.

*References in each subgroup were listed in forest plots of Appendix S7 in the **Online Supplementary Document**.

## DISCUSSION

In this study, we found that the NPIs, vaccinations, and treatments were all cost-effective. Among pooled NPIs, suppression and screening were associated with higher health and economic effect. Vaccinations were largely cost-effective and even cost-saving, regardless of the coverage and relatively lower efficacy, with one study [101] recommending consideration of prioritisation options. Ongoing COVID-19 treatment mainly focused on dexamethasone, remdesivir, and tocilizumab, and the results showed they were cost-effective and even cost-saving, especially in the long-time horizon.

To our best knowledge, this is the first study that systematically synthesizes the evidence on the cost-effectiveness of prevention and control interventions against COVID-19. Only one study previously reviewed this aspect [20] and provided an overview summary of the cost-effectiveness of programs against COVID-19 without pooling the effect size. By then, the cost-effectiveness of COVID-19 vaccinations had not been studied. Compared to the previous systematic review, we updated the evidence and added the meta-analysis of the cost-effectiveness of COVID-19 vaccinations. Furthermore, we pooled the INB to provide concrete evidence on the economic benefit of the interventions and made it easy to do subgroup analysis and explore the source of heterogeneity.

Among NPIs against COVID-19, suppression and screening offered higher economic efficiency than other interventions. Multiple interventions, including testing, isolation, and quarantine as mitigation strategies were committed to reducing peak health care demand and protecting those most at risk of severe disease from infection, while suppression and screening reversed epidemic growth and maintained that situation indefinitely [108]. We also found that higher cost-effectiveness of the intervention was found in high-income economies. Ferguson et al. [108] suggested that suppression was the preferred policy option for countries that could achieve it, which is consistent with our results and explains the source of heterogeneity. With the rapid transmission speed of new variants and in contexts of weaker health systems, along with the greater vulnerability of developing economies to the negative impact of stringent measures, the trade-offs faced by low-income countries are complex [106,109]. In addition, low-income states lack the infrastructure for implementing technology-led containment strategies [110]. However, with the success of movement restriction policies, isolation centres, quarantine centres, and contact tracing in China and South Africa, NPIs are still suggested as cost-effective options in dealing with the COVID-19 pandemic, at least at its onset [14,35].

Our results show that the benefits from a health perspective were significantly higher than from a social perspective. Zhao et al. [35] suggested that the results were most sensitive to the hospital costs per critical case, the number of days frontline health care workers worked, and the number of frontline health care workers from a health care perspective, while they were most sensitive to the number of non-infected employed persons, the national average wage per working day, and the number of hours worked by non-infected persons from a social perspective. The heterogeneity should be interpreted as a result of the NPIs contributing to alleviating the strain on health care resources and decreasing the high workload of the health care system and health care workers, while imposing a considerable burden on the public's productivity and life in low-and-middle-income economies.

All cost-effectiveness analyses of COVID-19 vaccinations were conducted for upper-middle- or high-income countries and showed that vaccinations would bring large economic benefits, regardless of the relatively lower efficacy, and should be initiated as soon as the first vaccine is available, and that coverage should be expanded. COVID-19 vaccination would still be beneficial overall, even if the vaccines have 50% efficacy and provide only short-lived protection against disease [91]. However, the prioritization and allocation of vaccines are still being debated. It is cost-saving and more effective when prioritizing individuals aged ≥65 for vaccination since they are at higher risk of hospitalization and death and have a greater need for maintaining social distance [89,90]. In the younger population with a tendency for higher contact rates, vaccination with relatively lower efficacy and great coverage could reduce the probability of transmission and would be cost-effective [101,111]. Du et al. [99] suggested that dose-sparing strategies could save a large number of lives, even with the emergence of new variants with higher transmissibility in India.

As for treatments of COVID-19, the pooled INB was significantly higher with supportive care when compared to standard care. Supportive care is diverse and involves multidisciplinary cross-collaboration, including but not limited to timely intubation, reasonable mechanical ventilation support, appropriate anti-infective therapy, early anticoagulation and immune support, and other comprehensive measures that help reduce the disease’s course and patient mortality [112]. The heterogeneity could be explained by the higher medical burden that supportive care carries when compared to standard care, so the treatment strategy is more cost-effective when it is used as a control. For group time horizon results, Sheinson et al. [39] have demonstrated that even small treatment-related alterations in the acute phase can lead to significant changes in QALYs and costs over a patient's lifetime, therefore the long-term economic benefits outweigh the short-term ones. However, given scarce resources in lower-income economies, essential and advanced critical care needed for the management of severe and critical COVID-19 were both lacking. Kairu et al. [106] found that Kenya could achieve better value-for-money if it prioritized investments in essential care before investments in advanced critical care.

The results of the sensitivity analysis were highly robust. According to the funnel plot and Egger’s test, publication bias might appear in the meta-analysis of NPIs and treatments against COVID-19, but the results of the trim-and-fill method suggested that treatments would still be cost-effective even if the six missing interventions were included. No missing studies were found in the funnel of NPIs, indicating the resource of symmetry might be high heterogeneity. All pooled INB were still positive even with one study excluded from the analysis. The failsafe number of three meta-analyses was less than that of the un-pooled study, indicating there is little chance that bias might exist in a partial meta-analysis.

The present review had several potential limitations. First, although we conducted a subgroup analysis to identify potential sources of heterogeneity and used a random-effects model, heterogeneity still existed among studies and interventions, which might lead to bias. However, we summarised the studies so we could make our final suggestions. By pooling INBs, we aimed to make the comparison more concrete. Second, our findings may be generalizable to only high or middle-income countries because all included studies were from those countries. Third, there might be publication bias and investigator bias in the literature as shown in funnel plot of NPIs. Although we adjusted for this by using the trim-and-fill method, the possible bias might still exist. Fourth, there are many challenges in synthesising economic evaluation studies as they differ in the method of reporting results and using economic parameters, and so on. We attempted to derive the suitable parameters for pooling by standardising the studies under different scenarios, which might be imprecise. However, our final conclusions were made based on both INB pooling and study summary to make them more solid.

## CONCLUSIONS

NPIs, vaccination, and treatment for COVID-19 are all cost-effective. Suppression and screening are more cost-effective than other NPIs. Our findings highlight the value of NPIs in addressing COVID-19 and emphasize the importance of advancing vaccine coverage in anticipation of controlling outbreaks and repairing and maintaining health system stability. More studies are needed to explore appropriate strategies and tools for intermediate and lower economies, thereby reducing the global burden of COVID-19 and returning the world to its previous order.

## Additional material


Online Supplementary Document
