# Supplementary material for: Cost-effectiveness of interventions for the prevention and control of COVID-19: Systematic review of 85 modelling studies
Source: J Glob Health. 2022 Jun 15;12:05022. doi: 10.7189/jogh.12.05022 (PMC9196831; doi:10.7189/jogh.12.05022)
Supplement: Online Supplementary Document [file jogh-12-05022-s001.pdf]

Supplementary materials

Contents

Appendix S1 Search strategies..2

Appendix S2 Methods for INB and its 95% CI calculation in different scenarios[1,2] 4

Appendix S3 Study design and setting overview 5

Appendix S4 Risk of bias assessments 31

Appendix S5 Funnel plots..32

Appendix S6 Sensitivity analyses 33

Appendix S7 Subgroup analyses 35

Reference 44

Appendix S1 Search strategies

Table S1 Search terms used in four databases

Database	Search strategy	Items found
Pubmed	(Cost[ti] OR "cost analysis"[tiab] OR (Analysis[tiab] AND Cost[tiab]) OR costing[tiab] OR "Cost Comparison"[tiab] OR cost-effectiveness[tiab] OR "cost effectiveness"[tiab] OR cost-utility[tiab] OR "cost utility"[tiab] OR cost-benefit[tiab] OR "cost benefit"[tiab] OR "economic evaluation"[tiab] OR "health resource allocation"[tiab] OR "Medical Economics"[ti] OR (economic[ti] AND medical[ti]) OR "health economics"[ti] OR economic*[ti] OR "decision analysis"[tiab] OR decision-analytic[tiab]) AND (covid-19[tiab] OR "2019 novel coronavirus disease"[tiab] OR "COVID 19"[tiab] OR "COVID-19 pandemic" [tiab] OR "SARS-CoV-2 infection"[tiab] OR "COVID-19 virus disease"[tiab] OR "2019 novel coronavirus infection"[tiab] OR "2019-nCoV infection"[tiab] OR "coronavirus disease 2019"[tiab] OR "coronavirus disease-19"[tiab] OR "2019-nCoV disease"[tiab] OR "COVID-19 virus infection"[tiab]) AND 2019/12/31:2022/3/22[dp]	2532
Embase	(Cost:ti OR "cost analysis":ab,ti OR (Analysis:ab,ti AND Cost:ab,ti) OR costing:ab,ti OR "Cost Comparison":ab,ti OR cost-effectiveness:ab,ti OR "cost effectiveness":ab,ti OR cost-utility:ab,ti OR "cost utility":ab,ti OR cost-benefit:ab,ti OR "cost benefit":ab,ti OR "economic evaluation":ab,ti OR "health resource allocation":ab,ti OR "Medical Economics":ti OR (economic:ti AND medical:ti) OR "health economics":ti OR economic*:ti OR "health resource allocation":ab,ti OR "decision analysis":ab,ti OR decision-analytic:ab,ti) AND (covid-19:ab,ti OR "2019 novel coronavirus disease":ab,ti OR "COVID 19":ab,ti OR "COVID-19 pandemic":ab,ti OR "SARS-CoV-2 infection":ab,ti OR "COVID-19 virus disease":ab,ti OR "2019 novel coronavirus infection":ab,ti OR "2019-nCoV infection":ab,ti OR "coronavirus disease 2019":ab,ti OR "coronavirus disease-19":ab,ti OR "2019-nCoV disease":ab,ti OR "COVID-19 virus infection":ab,ti) AND [2019-2022]/PY	2238
Web of Science	(TI=(Cost) OR TS= ("cost analysis") OR (TS= (Analysis) AND TS= (Cost)) OR TS= (costing) OR TS= ("Cost Comparison") OR TS= (cost-effectiveness) OR TS= ("cost effectiveness") OR TS= (cost-utility) OR TS= ("cost utility") OR TS= (cost-benefit) OR TS= ("cost benefit") OR TS= ("economic evaluation") OR TS=("health resource allocation") OR TI=("Medical Economics") OR (TI =(economic) AND TI=(medical)) OR TI=("health economics") OR TI= (economic*) OR TS=("decision analysis") OR TS=(decision-analytic)) AND (TS=(covid-19) OR TS=("2019 novel coronavirus disease") OR TS=("COVID 19") OR TS=("COVID-19 pandemic") OR TS=("SARS-CoV-2 infection") OR TS=("COVID-19 virus disease") OR TS=("2019 novel coronavirus infection") OR TS=("2019-nCoV infection") OR TS=("coronavirus disease 2019") OR TS=("coronavirus disease-19") OR TS=("2019-nCoV disease") OR TS=("COVID-19 virus infection")) AND PY=(2019-2022)	11841
Cochrane	#1 (Cost:ti OR "cost analysis":ab,ti OR (Analysis:ab,ti AND Cost:ab,ti) OR costing:ab,ti OR "Cost Comparison":ab,ti OR "health care cost":ab,ti OR cost-effectiveness:ab,ti OR "cost effectiveness":ab,ti OR cost-utility:ab,ti OR "cost utility":ab,ti OR cost-benefit:ab,ti OR "cost benefit":ab,ti OR "economic evaluation":ab,ti OR "health resource allocation":ab,ti "health economic":ab,ti OR (economic:ab,ti AND medical:ab,ti) OR pharmacoeconomic:ab,ti OR "decision analysis":ab,ti OR decision-analytic:ab,ti OR economic*:ti) with Publication Year	207

Database	Search strategy	Items found
	from 2019 to 2022, in Trials<br>#2 (covid-19 OR "2019 novel coronavirus disease" OR "COVID 19" OR "COVID-19 pandemic" OR "SARS-CoV-2 infection" OR "COVID-19 virus disease" OR "2019 novel coronavirus infection" OR "2019-nCoV infection" OR "coronavirus disease 2019" OR "coronavirus disease-19" OR "2019-nCoV disease" OR "COVID-19 virus infection") with Publication Year from 2019 to 2022, in Trials<br>#3 #1AND#2	

Appendix S2 Methods for INB and its 95% CI calculation in different scenarios[1,2]

Studies reported the mean and standard error of C (or ΔC) and E (or ΔE). We estimated the mean and variance of INB as the equation followed using Monte Carlo simulation for the times of sample size or 1000 if sample size was not reported:

$$\mathrm{INB} = \mathrm{K} \times \Delta\mathrm{E} - \Delta\mathrm{C}$$

$$\mathrm{var(INB)} = K^2{\sigma_{\Delta E}}^2 + {\sigma_{ICER}}^2$$

Studies reported the mean of C (or ΔC) and E (or ΔE), but the dispersions of these parameter was not reported. The cost and effect of studied and control interventions were extracted to estimate the mean and variance of ΔC and ΔE. Then, the mean of INB was calculated as the equation mentioned above, and the variance was estimated as follows:

$$\mathrm{var(INB)} = K^2{\sigma_{\Delta E}}^2 + {\sigma_{\Delta C}}^2 - 2\rho_{\Delta C\Delta E}$$

where $\rho_{\Delta C\Delta E}$ is the covariance of ΔC and ΔE.

For studies reporting the mean and 95% CI of C (or ΔC) and E (or ΔE), the variance was estimated as follows:

$$\mathrm{SE} = \frac{UL - \mu}{1.96}$$

where UL is the upper limit and μ is the mean. The INB was further calculated using the above equations.

Studies reported the mean and standard error of net monetary benefit (NMB) of the studied and control interventions. The INB and its variance was estimated by simulating the NMB for1000 replications and calculated by equation: $\mathrm{INB} = \mathrm{NMBs} - \mathrm{NMBc}$, where NMBs and NMBc were the NMB of the studied and control interventions respectively.

Studies reported only the means of outcome parameters without reporting the measures of dispersion. Nevertheless, the cost-effective plane graph for ΔC and ΔE was provided. Data for ΔC and ΔE could be then extracted from the graph using the WebPlotDigitizer software version 4.5. The 95% CI of INB could be derived then.

Appendix S3 Study design and setting overview

Table S2 Study design and setting overview.

Group and Pooled	Author	Country	Type of economic evaluation	Population	Perspective	Intervention	Comparator	Sensitivity analysis	Time horizons	Discount rate	Parameter index	WTP	Conclusion	CHEERS score
**Vaccine**														
No	Bartsch et al. (1) (2021)[3]	US	CEA (a computational model)	The general population	The third-party payer and societal	Increased vaccination coverage	Lower vaccination coverage	Yes	3 years	3%	ICER	$50000 /QALY	Cost-effective	0.67
No	Bartsch et al. (2) (2021)[4]	US	CEA (a computational model)	The general population	The third-party payer and societal	Vaccination when an initial vaccine is available	Late vaccination waiting for a vaccine with a higher efficacy	Yes	2.5 years	None	ICER	$50000 /QALY	Cost-effective	0.67
No	Hagens et al. (2021)[5]	Turkey	CEA (an enhanced SIRD model)	patients who have an	Health care system	Strategies of vaccination	No vaccination	Yes	1 year	3.00%	ICER	$9127 /QALY	Cost-effective	0.88

				indication for ICU care	and societal								even cost-saving	
No	Kirwin et al. (2021)[6]	Canada	CBA (the COVID-19 Risk Assessment Model, a transmission dynamic, ordinary differential equation, compartmental susceptible-exposed-infected-recovered model.)	The general population	Health system payer	Vaccination allocation strategies defined by age and risk target groups, coverage, effectiveness and cost	No vaccination	NR	1 October 2020-6 February 2021	1.50%	NMB	$30000 /QALY	Dependent on the primary outcome of interest	0.83
No	Kohli et al. (2021)[7]	US	CEA (a Markov cohort model)	Entire adult population	Health care system	A 60% efficacious vaccine.	No vaccination	Yes (DSA)	NR	3.00%	ICER	$50000 /QALY	Cost-effective	0.92
Yes	Marco-Franco et al. (2021)[8]	Spain	CEA (Markov model)	The general population	Buyer	Vaccination	No vaccination	Yes	27 October 2020-17	3.50%	ICER	€27500 /QALY	Cost-effective	0.92

									February 2021					
	Padula et al. (2020)[9]	US	CUA (SEIR model)	The general population	Societal	Vaccination	No vaccination	Yes (one-way and PSA)	1 year	3.00%	ICER	$100000 /QALY	Cost-effective	0.92
Yes	Reddy et al. (2)(2021)[10]	South Africa	CEA ((CEACOV) a dynamic state-transition Monte Carlo microsimulation model)	The general population	Health care system	Strategies of vaccination	No vaccination	Yes	360 days	NR	ICER	$3250 /LY	Cost-effective	0.75
No	Sandmann et al. (2021)[11]	UK	CBA (COVIDM, an age-stratified dynamic transmission model)	The general population	Health-care payer and the national economy	Vaccination	No vaccination	NR	10 years	3.50%	NMB	£20000 /QALY	Vaccination was cost-effective, increased physical distancing might not	0.75
Yes	Shaker et al. (2021)[12]	US	CEA (decision tree)	The general population	Health care system and societal	Universal vaccination	Risk-stratified vaccination	Yes (DSA and PSA)	1 year	NR	ICER	$10000000 /death prevented	Cost-effective	0.79

Yes	Wang et al. (2021)[13]	Israel	CUA (Markov decision tree)	The general population	Healthcare payer and societal	Vaccination (Moderna, Pfizer and AstraZeneca)	No vaccination	Yes (DSA and PSA)	180 days	NR	BCR	1	Cost-effective	0.79
No	López, F., et al (2021) [14]	Catalonia	CBA (an epidemiological model)	The general population	Healthcare payer and societal	The mass vaccination campaign against COVID	No vaccination	Yes	9 months	None	BCR	1	Cost-saving	0.79
No	Debrabant, K., et al (2021) [15]	Denmark	CEA (an epidemiological model)	The general population	Danish healthcare sector	(i) vaccination of 25% of the total population but targeting vaccines towards the population aged above 60 years, (ii)vaccination of 25% of the total population, targeting vaccines only towards the population aged <60 years, (iii)vaccination of 40% of the total population where 15% are aged <60 years and 25% are aged above 60 years, (iv) 40% of the total population is vaccinated but vaccines are targeted	Status quo	Yes	6 months	0%, 2% or 4%	ICER	-	The cost effectiveness of a COVID-19 vaccine is sensitive to whether or not productivity losses are included in the analyses.	0.96

						solely towards those aged <60 years.								
No	Pearson, C. A. B. et al (2021) [16]	Sindh Province, Pakistan	CEA (a compartmental transmission model)	The general population	The health system	Vaccination	No vaccination	Yes	30 April to 15 September 2020	3%	ICER	$500 per DALY averted	Cost-effective	0.88
No	Vaezi, A., et al (2021) [17]	Iran	CEA (a compartmental transmission model)	The general population	NR	Vaccination	No vaccination	Yes	NR	NR	ICER	2282 USD per DALY averted	Considering the scenario of Iran, vaccines that are recommended include ad26.cov2.s, chadox1-S, rAd26-S + rAd5-S, and BNT162b2 in the order of recommendation	0.69
No	Jiang, Y., et al (2022) [18]	Hong Kong SAR, Indonesi	CEA (decision tree)	The general population	Societal	Inactivated COVID-19 vaccines	No vaccination	Yes	1 year	3%	ICER	One time GDP per capita	Cost-effective	0.94

		a, mainland China, Philippines, Singapore, and Thailand												
No	Kirson, N., et al (2022) [19]	US	CBA	The general population	Societal	Vaccination	No vaccination	Yes	3.5 years	3%	INB	$150000/LY	Cost-effective	0.71
No	Du, Z. et al (2022) [20]	India	CEA (a multi-scale model)	The general population	Indian health sector	Strategies of vaccination	No vaccination	Yes	150 days	3%	NMB	$1,097-3205/averted YLL in different age group	Fractional dosing of vaccines is cost-effective	0.83
No	Liu, Y., et al (2022) [21]	The WHO European Region	CEA (age-specific compartmental models)	All adults	NR	COVID-19 vaccine prioritisation strategies	Each other	Yes	2 years	3%	Five decision-making metrics	-	Countries expecting a slow vaccine roll-out may particularly benefit from prioritising older adults.	0.77

No	Bartsch, Sarah M. et al. (2021) [22]	US	CEA (a computational model)		The third-party payer	Increasing vaccination coverage	No vaccination	Yes (DSA and PSA)	3 years	3.5%	NMB	-	Cost-effective	0.83
Yes	Li, R., et al (2021) [23]	US	CEA (a decision-analytic Markov model)	100,000 cohort of older adults (above 65 years)	healthcare system	booster strategy	2-doses of BNT162b2	Yes (DSA and PSA)	180 days	3%.	ICER	$50000/death averted	Cost saving	0.92
**Treat**														
Yes	Aguas et al. (2021)[24]	UK	CEA (simple state transition model)	Hospitalized COVID-19 patients	Health care system	Dexamethasone treatment	Standard care	Yes	July-December 2020	None and (a discrete-time discount factor)	ICER	£20000 /LY	Cost-effective	0.71
No	Cleary et al. (2021)[25]	South Africa	CEA (A decision analytic model)	Hospitalized COVID-19 patients	Societal	General ward and intensive care (GW + ICU)	General ward only	Yes	From admission to discharge or death	None	ICER	ZAR 38465 /DALY averted	Not cost-effective	0.94

No	Gandjour et al. (2021)[26]	Germany	CEA (a life-table model)	COVID-19 patients with an indication for ICU care	Societal	The provision of additional capacity of ICU	No intervention	Yes (DSA)	Life time	Applied in sensitivity analysis	Marginal cost-effectiveness ratio	€ 101493 /life year gained	Cost-effective	0.85
No	Jiang et al. (2021)[27]	China	CEA (A dynamic transmission model extended from SEIR)	Hospitalized COVID-19 patients	Health care system	Remdesivir treatment	Standard care	Yes	55 days and life time	5.00%	ICER	￥70892 /QALY	Cost-effective	0.90
Yes	Jo et al. (2020)[28]	South Africa	CEA (the South African National COVID-19 Epi Model.)	COVID-19 patients in ICU	Health care system	Dexamethasone or remdesivir	Standard care	Yes	August 2020-January 2021	5.00%	ICER	$1000 /death averted	Cost-effective even cost-saving	0.83
Yes	Sheinson et al. (2021)[29]	US	CEA (A lifetime model, with a decision tree followed by a Markov cohort model)	Hospitalized COVID-19 patients	Health payer, societal, and fee-for-service (FFS) payment	The average between drug treatment arm	Best supportive care	Yes (DSA and PSA)	Life time	3.00%	ICER	$100000~150000 /QALY	Cost-effective	0.88

Yes	Sinha et al. (2021)[30]	UK	CEA (decision tree)	Severe COVID-19 patients	NR	Tocilizumab and Dexamethasone	Dexamethasone alone	Yes (DSA and PSA)	Life time	3.00%	ICER	$100000 /QALY	Cost-effective	0.88
Yes	Carta, A. et al [31]	US	CEA (A decision tree)	Covid-19 hospitalized patients	Health care	Remdesivir, Dexamethasone and a simultaneous use of the two drugs	Standard care	Yes (DSA and PSA)	1 year	NR	ICER	$50000/QALY	Cost saving or cost effective	0.88
Yes	Rafia, R., et al [32]	England and Wales	CEA (A decision-analytical model)	Hospitalized patients requiring supplemental oxygen at the start of treatment	Health services	Remdesivi	Standard care	Yes (DSA and PSA)	Life time	None	ICER	£20000/QALY	Cost-effective	0.9
Yes	Oksuz, E., et al [33]	Turkey	CEA (A cost-effectiveness model)	COVID-19 patients hospitalized with < 94% saturation and low-flow oxygen therapy	The payer's	Remdesivi	Standard care	Yes	A COVID-19 episode time	None	ICER	$25797/QALY	Cost saving	0.92

				(LFOT) requiremen										
Yes	Ohsfeldt, R., et al [34]	US	CEA (A pharmacoeconomic model)	Hospitalized patients aged ≥18 years with COVID-19	Payor and hospital	Baricitinib	Standard care	Yes (DSA and PSA)	Life time	3.00%	ICER	$50000/QALY	Cost-effective	0.94
No	Jovanoski, N., et al [35]	US	CEA (A decision-analytical model)	Ambulatory patients with mild to moderate COVID-19	US payer	Casirivimab/imdevimab	Usual care	Yes	Life time	3%.	ICER	$100000/QALY	Cost-effective for most ambulatory patients with COVID-19	0.77
Yes	Kelton, K., et al (2021) [36]	US	CEA (A three-state model)	All COVID-19 patients and patients who required oxygen	Payor and hospital	Baricitinib–remdesivir combination	Remdesivi	Yes (DSA and PSA)	Life time	3%.	ICER	$50000/QALY	Cost-effective	0.94

Yes	Whittington, M. D., et al (2022) [37]	US	CEA (A Markov model)	Hospitalized patients with COVID-19	US healthcare sector	Remdesivi	Standard care	Yes (DSA and PSA)	Life time	3%.	ICER	$100000/QALY	Not cost-effective	0.96
No	Congly, S. E., et al (2021) [38]	US	CEA (A decision-analytical model)	60-year-old patient admitted to hospital with COVID-19	Health care system	Remdesivir to all patients, remdesivir in only moderate and only severe infections, dexamethasone to all patients, dexamethasone in severe infections, remdesivir in moderate/dexamethasone in severe infections, and best supportive care.	Best supportive care	Yes (DSA and PSA)	1 year	None	ICER	$100000/QALY	Dexamethasone for moderate-severe COVID-19 infections was the most cost-effective strategy, remdesivir not cost-effective	0.73
No	Kairu, A., et al [39]	Kenya	CEA (A decision tree)	COVID-19 patients	Health system	Investment in essential care and investment in both essential and advanced critical care	Current healthcare provision capacity (status quo)	Yes (DSA and PSA)	Inpatient care episode	3.00%	ICER	$908/DALY averted	Kenya should prioritise investments in EC before investments in ACC	0.88

No	Krylova, O., et al (2021) [40]	Russia	CEA (A decision tree)	COVID-19 patients	NR	Umifenovir/Remdesivir/Favipiravir	No intervention	NR	NR	NR	CER	-	favipiravir is optimal	0.75
**NPIs**														
No	Aldila et al. (2020)[41]	Indonesia	CEA (a deterministic model)	The general population	NR	Applying media campaign/rapid testing	No intervention	NR	NR	NR	ACER	NR	Cost-effective	0.63
No	Asamoah et al. (2020)[42]	Ghana	CEA (mathematical and optimal control model)	The general population	NR	effective testing and quarantine when boarders are opened, the usage of masks and face shields through media education, cleaning of surfaces with home-based detergents, practising proper cough etiquette and fumigating commercial areas	Each other	Yes	90 days	NR	ICER	NR	Safety measures (proper coughing etiquette) was the most cost-effective strategy	0.54
Yes	Bagepally et al. (2021)[43]	India	CEA (Markov model)	The general population	Public payers	hand-hygiene, surgical-mask N-95 respirators and surgical mask in general population	No intervention (& hand-hygiene)	Yes (DSA and PSA)	1 year	None	ICER	INR142719 /QALY	Hand hygiene was cost-effective and others were not	0.79
No	Baggett et al. (2020)[44]	US	CEA (dynamic	Adults residing in	Health care system	Daily symptom screening, universal PCR testing every 2 weeks, hospital-	No intervention	Yes (DSA)	April-August 2020	NR	ICER	$1000 /case prevented	Daily symptom screening	0.90

			microsimulation model)	homeless shelters		based or alternative care sites based COVID-19 care, and temporary housing							and ACSs was cost-effective (adding universal PCR testing every 2 weeks in surging epidemic)	
No	Barnett-Howell et al. (2021)[45]	178 countries	CBA (SEIR model)	The general population	NR	Social distancing and lockdowns	No intervention	NR	250 days	NR	VSL/GDP	NR	Not cost-effective	0.65
Yes	Broughel et al. (2021)[46]	US	CBA	The general population	NR	Suppression policies	No intervention	NR	NR	5%	NMB	$338000 /death averted	Cost-effective	0.56
No	Cook et al. (2021)[47]	Western Australia	CBA (SIR model)	The general population	Societal	Suppression policies and herd immunity	No intervention	Yes	1 year	NR	BCR	1	Not cost-effective	0.79
Yes	Du et al. (2021)[48]	US	CBA (a stochastic individual-based chain-binomial model)	The general population	Societal	Extensive SARS-CoV-2 testing & isolation	Symptom-based screening	Yes	150 days	A discrete-time discount factor	NMB	$100000 /YLL averted	Cost-effective	0.69

No	Ebigbo et al. (2021)[49]	NR	CEA (A Monte Carlo simulation)	Asymptomatic patients	NR	Pre-endoscopic virus testing and use of high-risk personal protective equipment	No intervention	Yes	NR	NR	ICER	NR	cost-effective with rising prevalence rates of COVID-19	0.50
No	Gandjour et al. (2020)[50]	Germany	NR (a life-table model)	The general population	Societal	A shutdown of businesses	No intervention	Yes	Life time	NR	Value of LYs gained	€ 101493 /life year gained	Cost-effective	0.65
No	Jiang et al. (2020)[51]	China	CEA (a deterministic compartment model extended from SEIR)	Hospitalized COVID-19 patients about to discharge	Health care system	Three (RT-PCR) tests for diagnosing and discharging people with COVID-19	Two (RT-PCR) tests	Yes	23 January 2020–6 March 2020 and life time	5.00%	NMB	￥64664 /QALY	Cost-effective	0.85
No	Khajji et al. (2020)[52]	Multi-region	CEA (a multi-region discrete mathematical model)	The general population	NR	WHO recommanded strategies (control of protecting susceptible individuals, preventing their contact with the infected individuals and encouraging the exposed individuals to join quarantine centers)	Each other	NR	NR	NR	ICER	NR	Cost-effective	0.50

No	Kouidere et al. (2021)[53]	Brazil	CEA (a mathematical model)	The general population	NR	Preventive and proactive measures (sensitization and prevention, diagnosis and treatment, quarantine and treatment, quarantine and diagnosis and monitoring with treatment)	Each other	NR	NR	NR	ICER	NR	Awareness campaigns to protect potential individuals infected with the virus, prevent contact with people infected with COVID-19 and with hospital quarantine for the infected is the most effective strategy	0.67
No	Lim et al. (2020)[54]	Singapore	CBA (branching process	The general population	NR	Expanded screening	No intervention	Yes	NR	None	NMB	NR	Cost-saving	0.58

			infection model)										
No	Lopez et al. (2021)[55]	Spain	CBA (López-Valcárcel and Vallejo-Torres model)	The general population	NR	Mass screening	Not mentioned	Yes	NR	3.00%	BCR	1	Cost-effective	0.71
No	Losina et al. (2021)[56]	US	CEA ((CEACOV) dynamic microsimulation model)	Undergraduate students and faculty at colleges.	Modified societal	COVID-19 mitigation strategies, including social distancing, masks, and routine laboratory screening.	No intervention	Yes	One semester (105 days)	NR	ICER	$100000 /QALY	Extensive social distancing with a mandatory mask-wearing policy was cost-effective, routine laboratory testing was not	0.83
No	Miles et al. (1) (2021)[57]	UK	CEA	The general population	NR	Lockdown	No intervention	NR	NR	NR	ICER	£30000 /QALY	Not cost-effective	0.58
No	Miles et al. (2) (2021)[58]	UK	CEA (SIR model)	The general population	NR	Easing in restrictions	Continuing lockdown	Yes	6 months	NR	NMB	£30000 /LY	Cost-effective	0.65

Yes	Neilan et al. (2020)[59]	US	CEA (a dynamic state-transition microsimulation model)	The general population	Health care system	hospital-based, symptom-based, asymptomatic screening	Symptom-based screening	Yes	180 days	3.00%	ICER	$100000 /QALY	Cost-effective	0.85
No	Newbold et al. (2020)[60]	US	CBA (discrete-time SIR compartment model)	The general population	NR	Physical distancing	No intervention	NR	NR	3.00%	NMB	$4500000 ~10000000 /VSL	Cost-effective	0.54
No	Omame et al. (2021)[61]	Nigeria	CEA (a mathematical model)	The general population	NR	Prevention among comorbid susceptibles, case detection control, control against reinfection,	Each other	Yes	200 days	NR	ICER	NR	Prevention among comorbid susceptibles was most cost-effective	0.54
Yes	Paltiel et al. (2020)[62]	US	CEA (A simple compartmental epidemic model)	Students in residential college setting	NR	SARS-CoV-2 screening	Symptom-based screening	Yes	80 days	NR	ICER	$5500~11600 /infection averted	Screening every 2 days using a rapid, inexpensive, and even poorly sensitive (>70%) test,	0.73

													coupled with strict behavioral interventions to keep Rt less than 2.5 was cost-effective	
Yes	Paltiel et al. (2021)[63]	US	CEA (A simple compartmental epidemic model)	The general population	Societal	Home-based SARS-CoV-2 antigen testing	No interventio n	Yes	60 days	NR	ICER	$5000000 ~17000000 /VSL	Cost-effective	0.81
Yes	Reddy et al. (1) (2021)[64]	South Africa	CEA ((CEACOV) a dynamic state-transition Monte Carlo microsimulation model)	The general population	Health care system	Contact tracing, isolation centres, quarantine centres	Health-care testing alone	Yes (DSA)	360 days	3.00%	ICER	$3250 /LY	Cost-effective	0.85
No	Risko et al. (2020)[65]	193 countries	CEA (a decision-analytic model with Bayesian multivariate	Health care workers	Societal	Adequate protection	Inadequate prtection	Yes (DSA and PSA)	30 weeks	NR	ICER	NR	Cost-effective	0.79

			sensitivity analysis and Monte Carlo simulation)											
No	Savitsky et al. (2020)[66]	US	CEA (Decision tree)	Health care workers	NR	Universal COVID-19 screening	Universal PPE use	Yes (one-way, two-way and PSA)	NR	None	ICER	$25000 /infection averted	Universal COVID-19 screening is generally the preferred option. However, universal PPE may be cost-effective and preferred in locations with high COVID-19 prevalence.	0.75
No	Scherbina et al. (2021)[67]	US	CBA (SIR model)	The general population	NR	Nation-wide lockdown	No intervention	Yes	2 March-8 March 2021	3.00%	NMB	$150000 /QALY	Cost-effective	0.58

No	Schonberger et al. (2020)[68]	US	CBA (Penn baseline model)	The general population	NR	Limited reopening with social distancing	Full Reopening & Reduced Social Distancing	Yes	NR	3.00%	NMB	$125000 /QALY	Cost-effective	0.69
No	Shlomai et al. (2021)[69]	Israel	CEA (A modified SEIRD model)	The general population	NR	A nationwide lockdown	Testing, tracing, and isolation	Yes (PSA)	200 days	0	ICER	$100000 ~150000 /QALY	Cost-effective	0.88
No	Thunstrom et al. (2020)[70]	US	CBA (SIR model)	The general population	NR	Social distancing	No intervention	Yes (break even analysis)	30 years	3.00%	NMB	NR	Uncertain and conditional	0.60
Yes	Zala et al. (2020)[71]	UK	CUA, CBA (individual-based simulation model and a macroeconomic model)	The general population	National health service	Suppression policies	No intervention	Yes (DSA)	NR	NR	ICER	£10000~70000 /QALY	Uncertain and conditional	0.54
Yes	Zhao et al. (2021)[72]	China	CEA (a dynamic simulation model)	The general population	Health care system and societal	Delayed movement restriction policies	Current practice	Yes (DSA and PSA)	NR	3.00%	NMB	￥70892 /DALY averted	Early implementation of movement	0.88

													restriction policies	
No	Gandjour, A. (2022) [73]	Germany	CEA (mathematical model with a closed form solution)	The general population	Consumer's perspective	COVID-19 self-tests	No self-tests	Yes	less than 1 year	NR	ICER	-	Cost-effective with minor benefit	0.81
No	Lally, M. (2022) [74]	Australia	CEA	The general population	NR	Australia's Covid-19 lockdown strategy	A mitigation strategy	NR	1 month	NR	ICER	$100000/QALY	Not cost-effective	0.6
No	Du, Z., et al (2022) [75]	US	CEA (a stochastic agentbased model)	Population in a typical US community	NR	Mass proactive testing and case isolation	A status quo testing strategy	Yes	150 days	NR	Cost and YLL averted	$100000/averted YLL	Cost-effective	0.88
No	Wang, X., et al (2022) [76]	The Tokyo 2020 Olympic Games and the upcoming Beijing 2022 Olympic	CEA (An agent-based stochastic dynamic model)	The competition-related personnel	NR	The COVID-19 surveillance strategies with different nucleic acid test frequancy	Strategy with lower nucleic acid test frequancy	Yes	NR	None	ICER	-	Increasing NAT frequency was cost-effective	0.9

		Winter Games.												
No	de Assis, T. S. M., et al (2022) [77]	Brazil	CEA (an analytical decision model based on decision trees)	Adult patients clinically suspected of COVID-19 after 14 days of symptoms onset, admitted at an outpatient or inpatient health care service	The Brazilian Unified Health System	Anti-SARS-CoV-2antibody diagnostic tests	Each other	Yes	The interval from the clinical suspicion of COVID-19 and serological test request until the outcome assessment, in this case, the test result.	NR	ICER	US$ 2.52/ properly diagnosed case	LFA tests are more cost-effective for estimated low-COVID-19-prevalences, while ELISAs are more cost-effective for high-pretest-probability scenarios	0.85
No	Zafari, Z., et al (2021) [78]	Columbia	CEA (a decision-analytic model)	Students, staff/faculty in Columbia University	NR	Interventions according to the CDC guidelines in combination with 1) a symptom-checking mobile application,	CDC guidelines	Yes (DSA and PSA)	90 days	3%	ICER	-	Cost-effective, only standardized, high	0.83

						2) university-provided standardized, high filtration masks,<br>3) thermal cameras for temperature screening,<br>4) one-time entry (‘gateway’) polymerase chain reaction (PCR) testing,<br>5) weekly PCR testing							filtration masks clearly provided value when prevalence of actively infectious cases in the community was low	
No	Wang, Q., et al (2021) [79]	China	CEA (A stochastic agentbased model)	The general population	NR	(1) program A: personal protection and isolationand- quarantine;<br>(2) program B: personal protection and community containment.	No interventio n	Yes	NR	None	ICER	-	Cost saving ot cost-effective	0.73
No	Suwantik a, A. A., et al (2022) [80]	Indonesi a	CEA (A modified Susceptible-Exposed-Infected-Recovered (SEIR) compartmenta l model)	The general population	Societal	Social distancing measures	No interventio n	Yes	1 year	NR	Cost and YLL averted	-	Cost-saving	0.81

No	Maya, S. et al (2022) [81]	US	CEA (a decision model)	Health care workers	NR	(1) only PCR test, (2) only Ag test (3) only IgG test, (4) conditional PCR test if IgG test is positive, and (5) concurrent IgG and PCR tests.	no tests	Yes (DSA and PSA)	NR	3%	ICER	-	Both PCR and antigen testing are beneficial strategies	0.83
No	Asamoah , J. K. K., et al (2022) [82]	Saudi Arabia	CEA (a non-autonomous nonlinear deterministic model)	The general population	NR	Practising physical or social distancing protocols, practising personal hygiene by cleaning contaminated surfaces with alcohol based detergents, practising proper and safety measures by exposed, asymptomatic infected and symptomatic infected individuals, fumigating schools in all levels of education, sports facilities, commercial areas and religious worship centres	Each other	Yes	100 days	NR	ICER	-	Strategy 1 (practising physical or social distancing protocols) is the most costsaving and most effective control intervention	0.63
No	Shimul, S. N. et	Saudi Arabia	CEA (The SIR model)	The general population	NR	Lockdown continued	Lockdown lifted	NR	NR	NR	ICER	$ 378 000/LY	Not cost-effective	0.65

	al (2021) [83]													
Yes	Kazungu, J., et al (2021) [84]	Kenya	CEA (a decision-analytic model)	Healthcare workers	Health system	Adequate/full Personal Protective Equipment (PPE) utilisation	Inadequate supply of PPE	Yes (PSA)	1 year	None	INB	$170.64 millions	Cost-effective	0.9
No	Bilinski, A., et al (2021) [85]	US	CEA (an agent-based network model)	Studutents and staff in a simulated elementary school and a middle school	Societal	The “test to stay” strategy	No school-based testing	Yes	30 days	NR	ICER	Elementary school: $4,000-20,000/infection directly averted	Cost-effective	0.69
No	Stevenson, M. et al (2021) [86]	UK	CEA (an agent-based network model)	A hypothetical hospital with an accident and emergency department	UK NHS	Viral detection point-of-care tests for detecting SARS-CoV-2	Laboratory-based tests	Yes	3 months	3.50%	ICER	-	Cost-effective	0.75
No	Thom, H., et al (2021) [87]	the UK, Ireland, German	CEA (Mathematical Modelling of	The general population	UK healthcare payer	Mitigation strategy	No mitigation	Yes	1 January to 20	NR	INB	£215.3-1907.6	Cost-effective	0.75

	y, Spain and Sweden	Infectious Diseases (CMMID) Covid-19 model)	July 2020

Abbreviations: NR: Not reported, CBA: Cost-benefit analysis, CUA: Cost-utility analysis, CEA: Cost-effectiveness analysis, CEACOV: Clinical and Economic Analysis of COVID-19 interventions, SIR: Susceptible, infectious, and recovered, SEIR: Susceptible, exposed, infectious, and recovered, SEIRD: Susceptible, exposed, infectious, recovered, and deceased, DSA: Deterministic sensitivity analysis, PSA: Probabilistic sensitivity analysis, USA: Univariate sensitivity analysis, RT-PCR: Reverse transcription polymerase chain reaction, ICER: Incremental cost-effectiveness ratio, ACER: Average cost-effectiveness ratio, VSL/GDP: Value of statistical life/ Gross domestic product, NMB: Net monetary benefit, BCR: Benefit cost ratio, QALY: Quality-adjusted life year, DALY: Disability adjusted life year.

Appendix S4 Risk of bias assessments

A

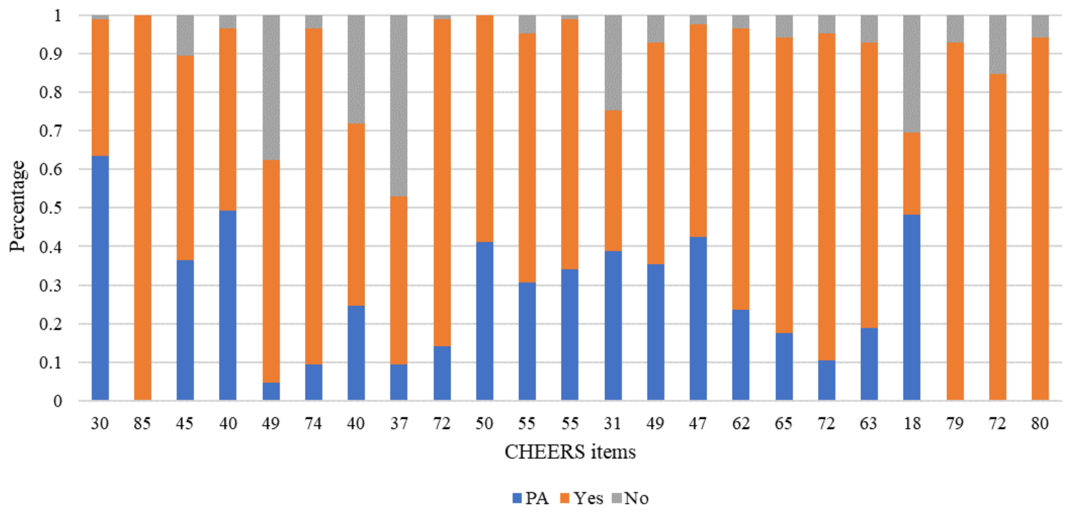

B

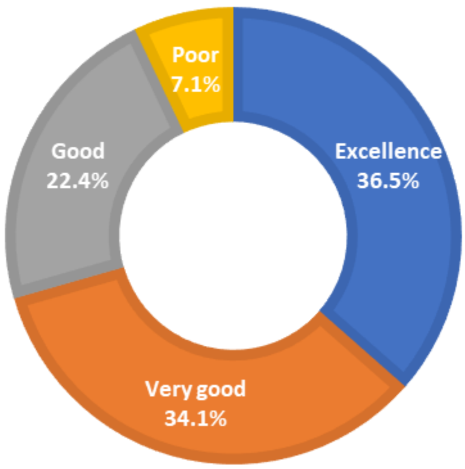

Figure S1 Reporting quality of included economic evaluation studies by CHEERS checklist. PA = part applied.

Appendix S5 Funnel plots

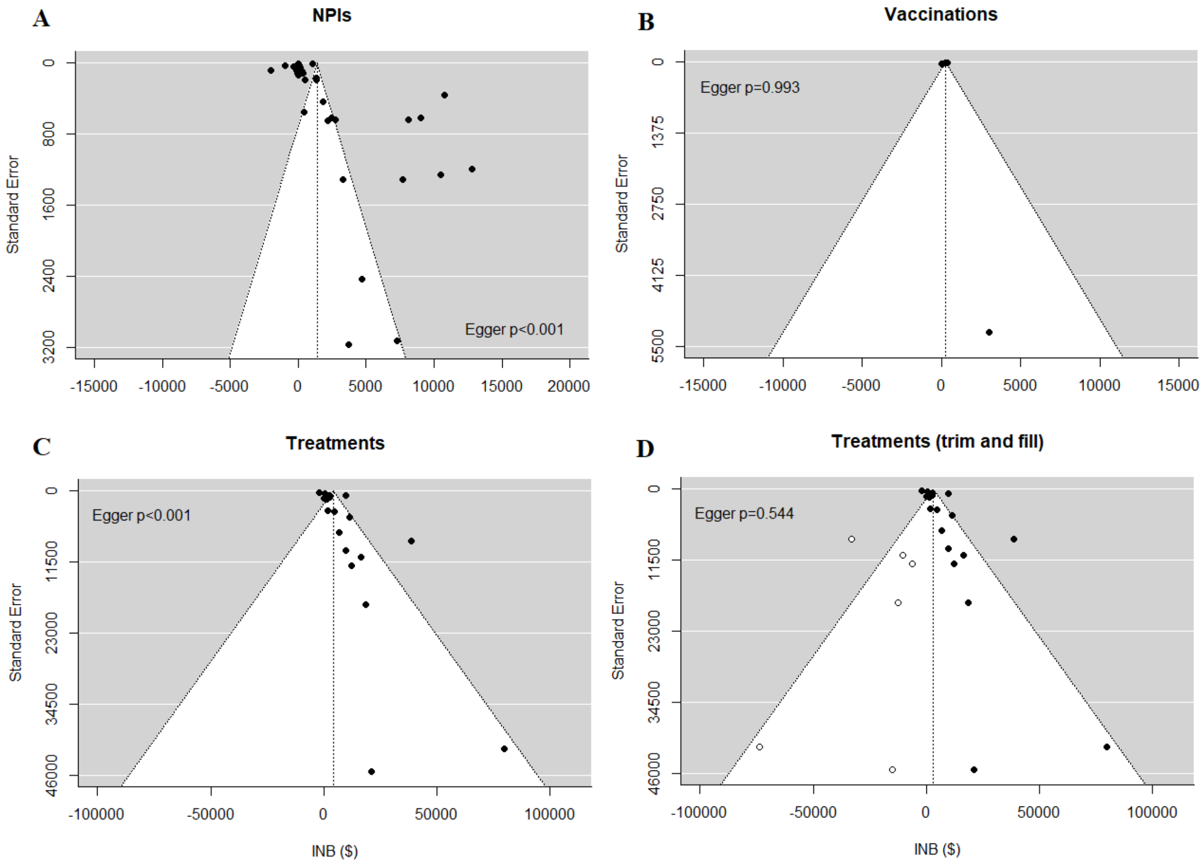

Figure S2 Funnel plot with pseudo 95% confidence limits of meta-analysis.

Appendix S6 Sensitivity analyses

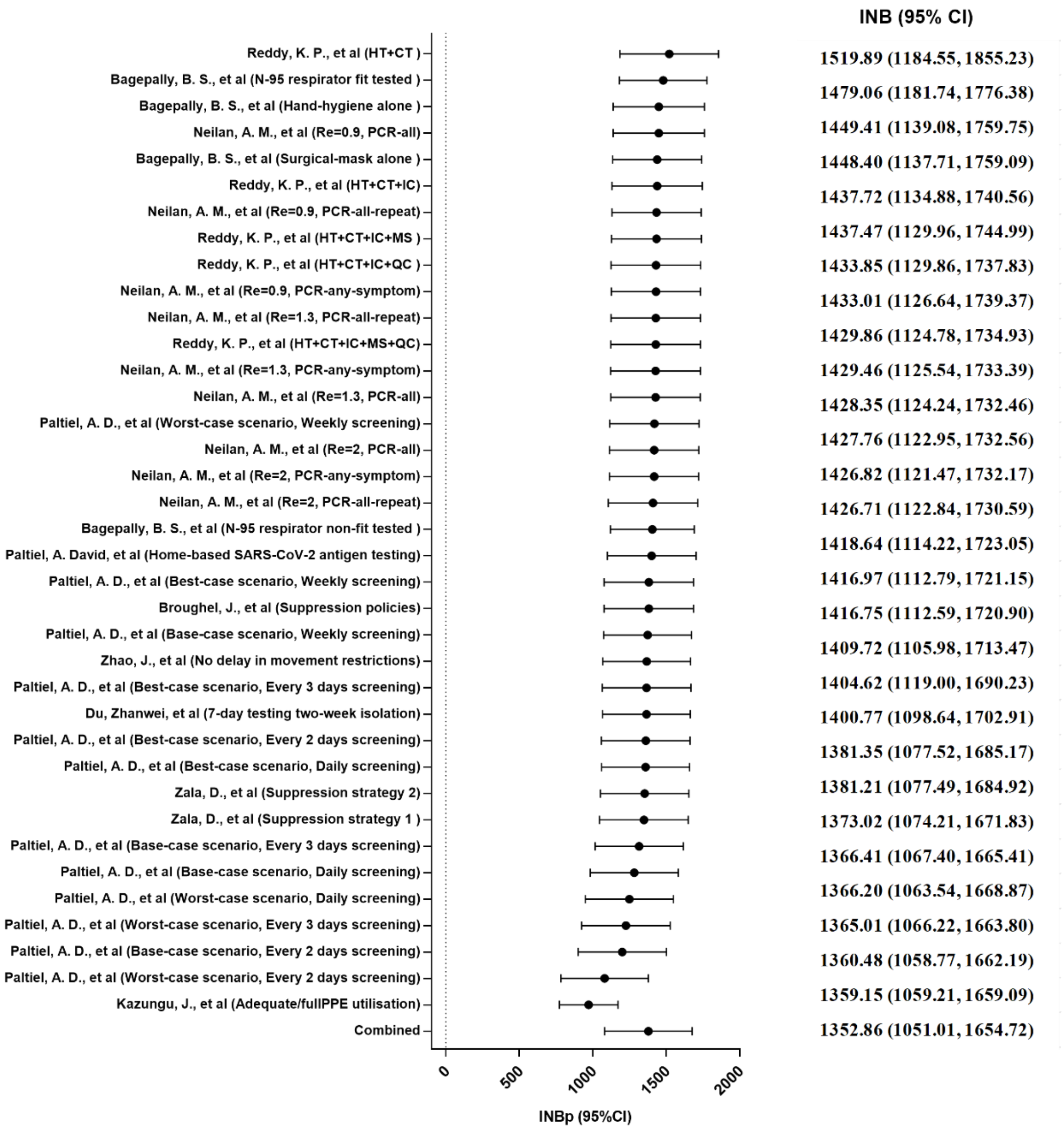

Figure S3 One study out influence analysis of the INB meta-analysis of NPIs of covid-19

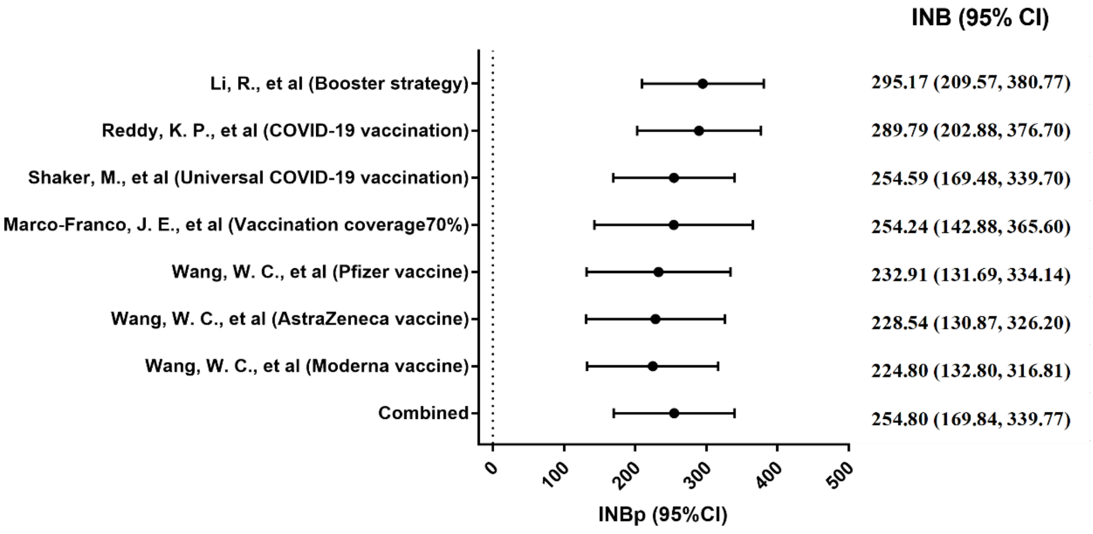

Figure S4 One study out influence analysis of the INB meta-analysis of covid-19 vaccinations.

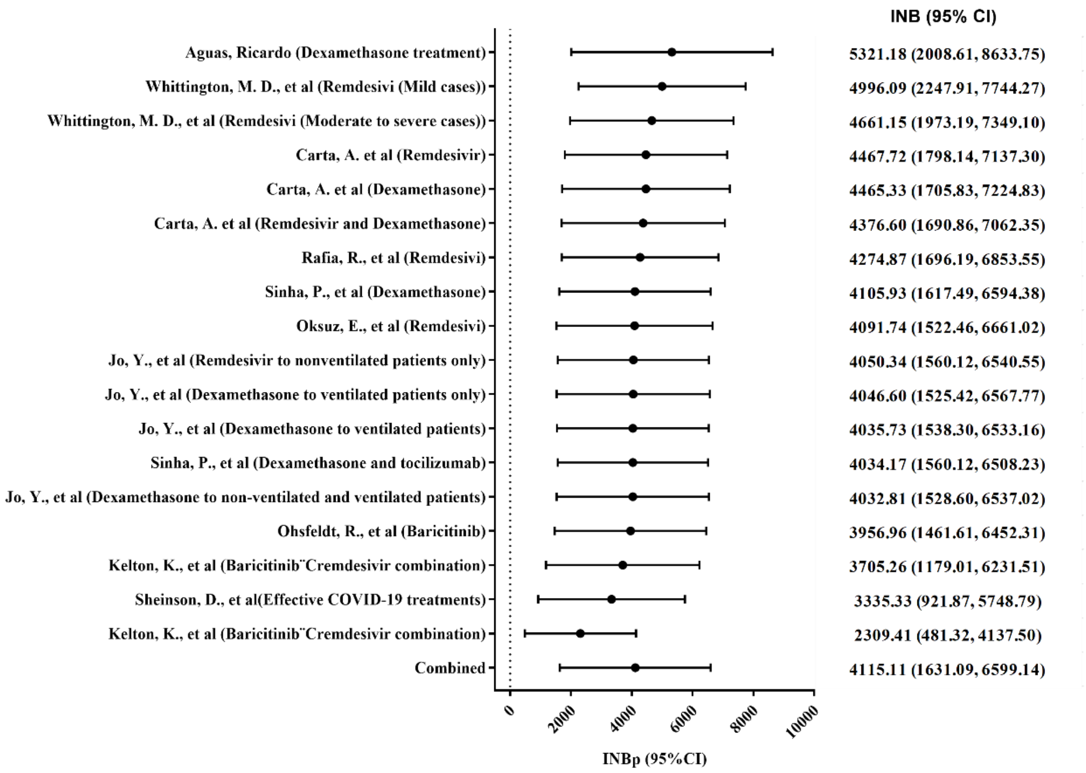

Figure S5 One study out influence analysis of the INB meta-analysis of treatments of covid-19.

Appendix S7 Subgroup analyses

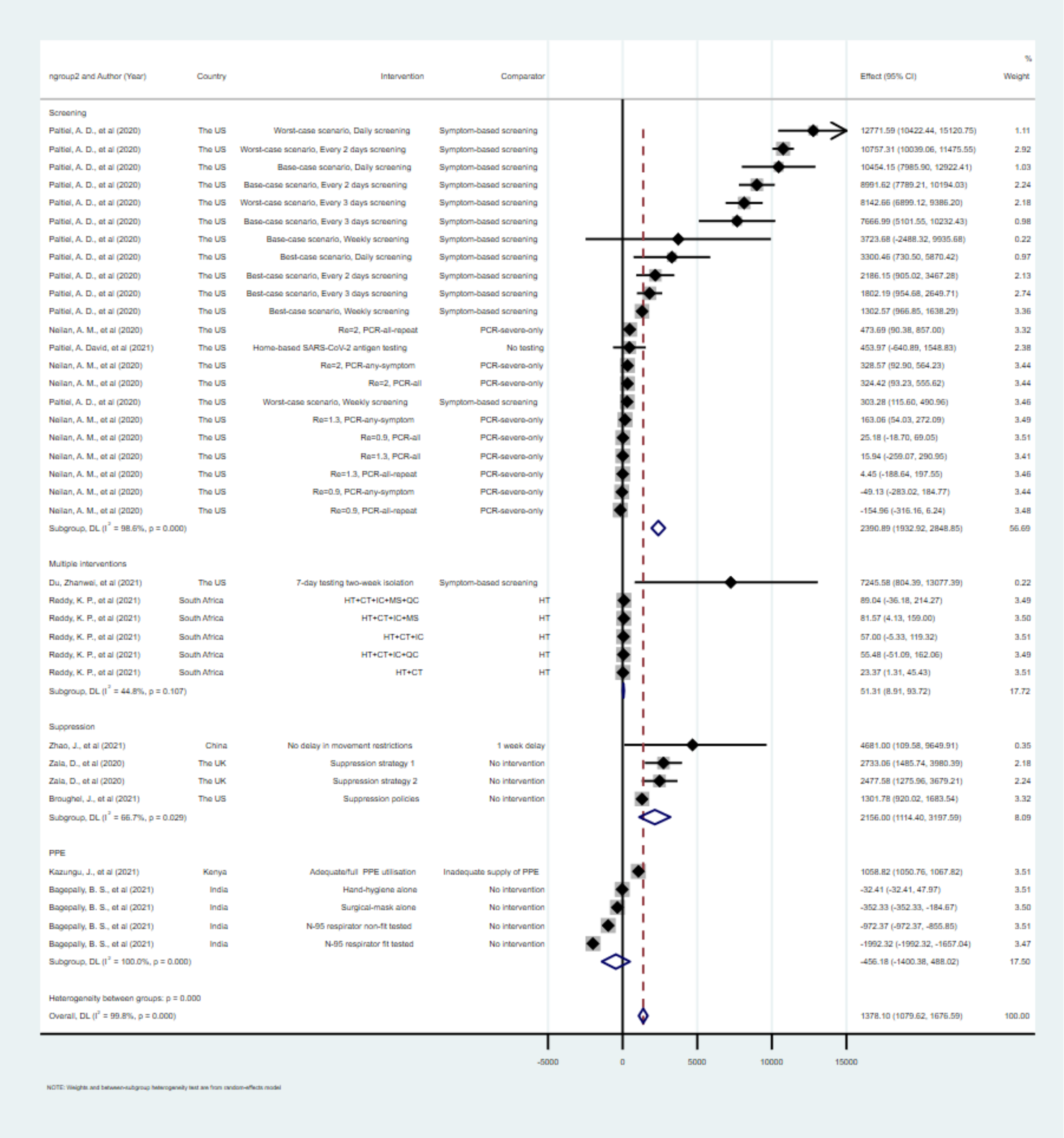

Figure S6 Subgroup analysis of the INB meta-analysis of NPIs of covid-19 in different intervention group.

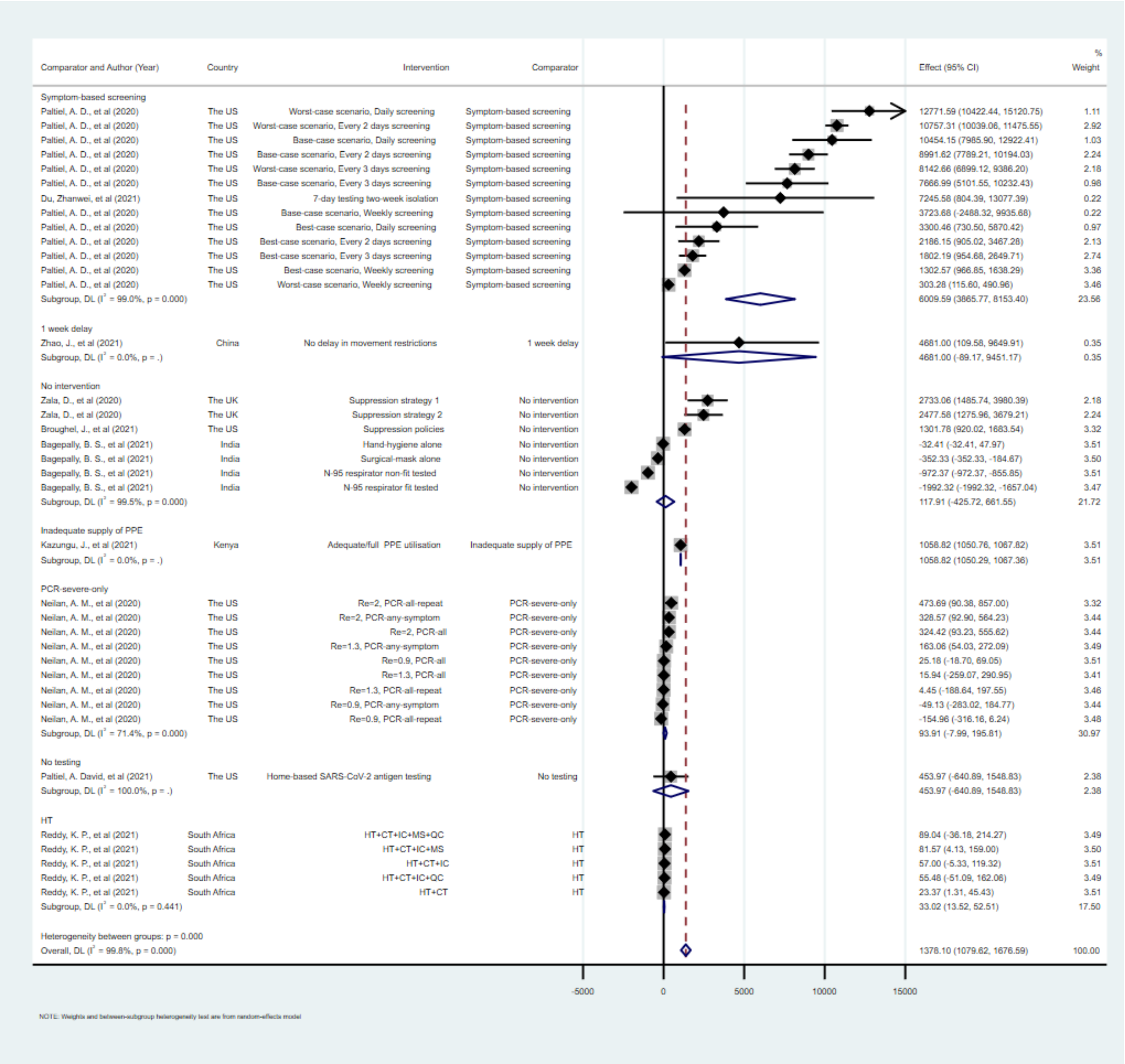

Figure S7 Subgroup analysis of the INB meta-analysis of NPIs of covid-19 in different comparator group.

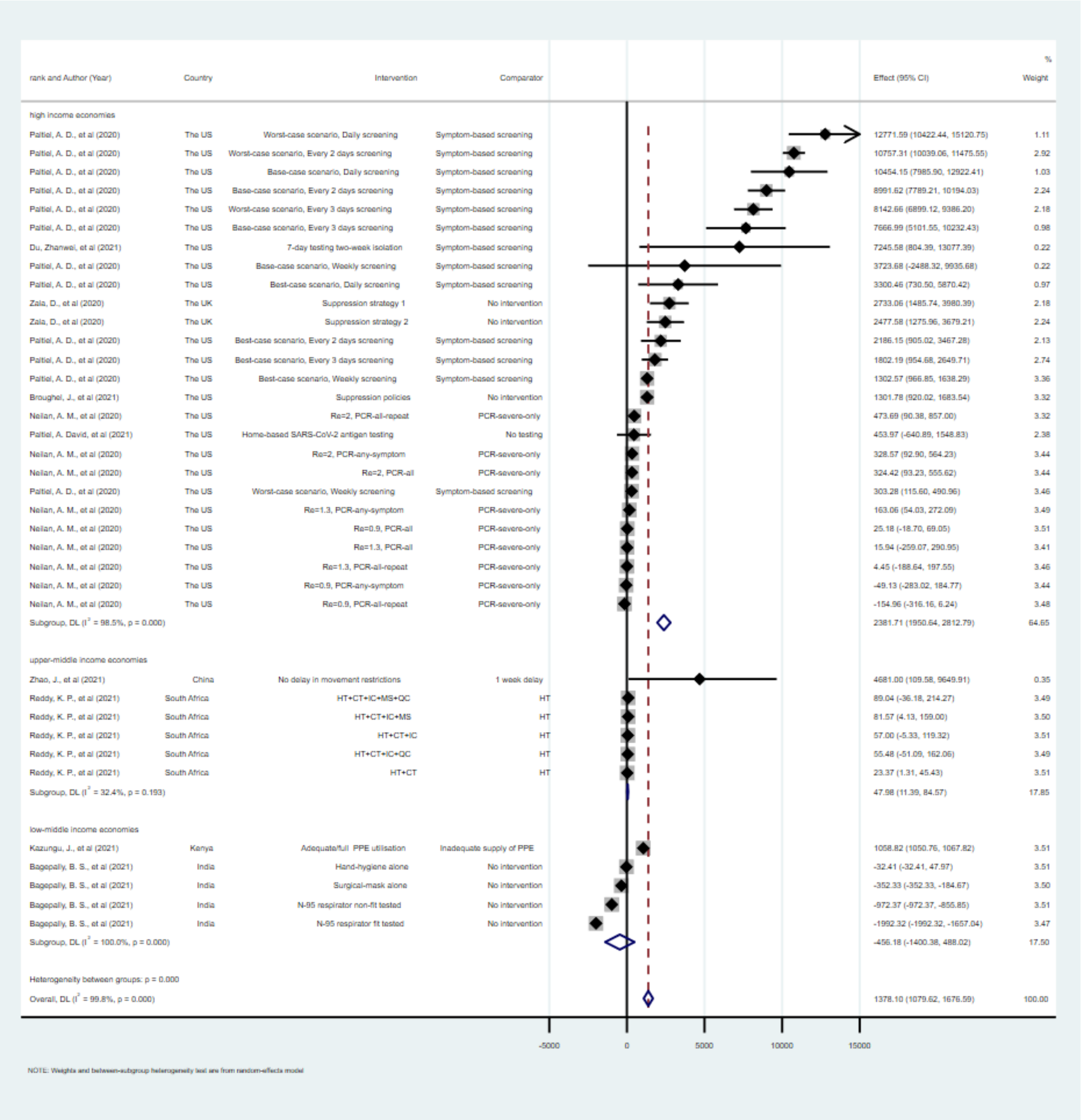

Figure S8 Subgroup analysis of the INB meta-analysis of NPIs of covid-19 in different income of economy group.

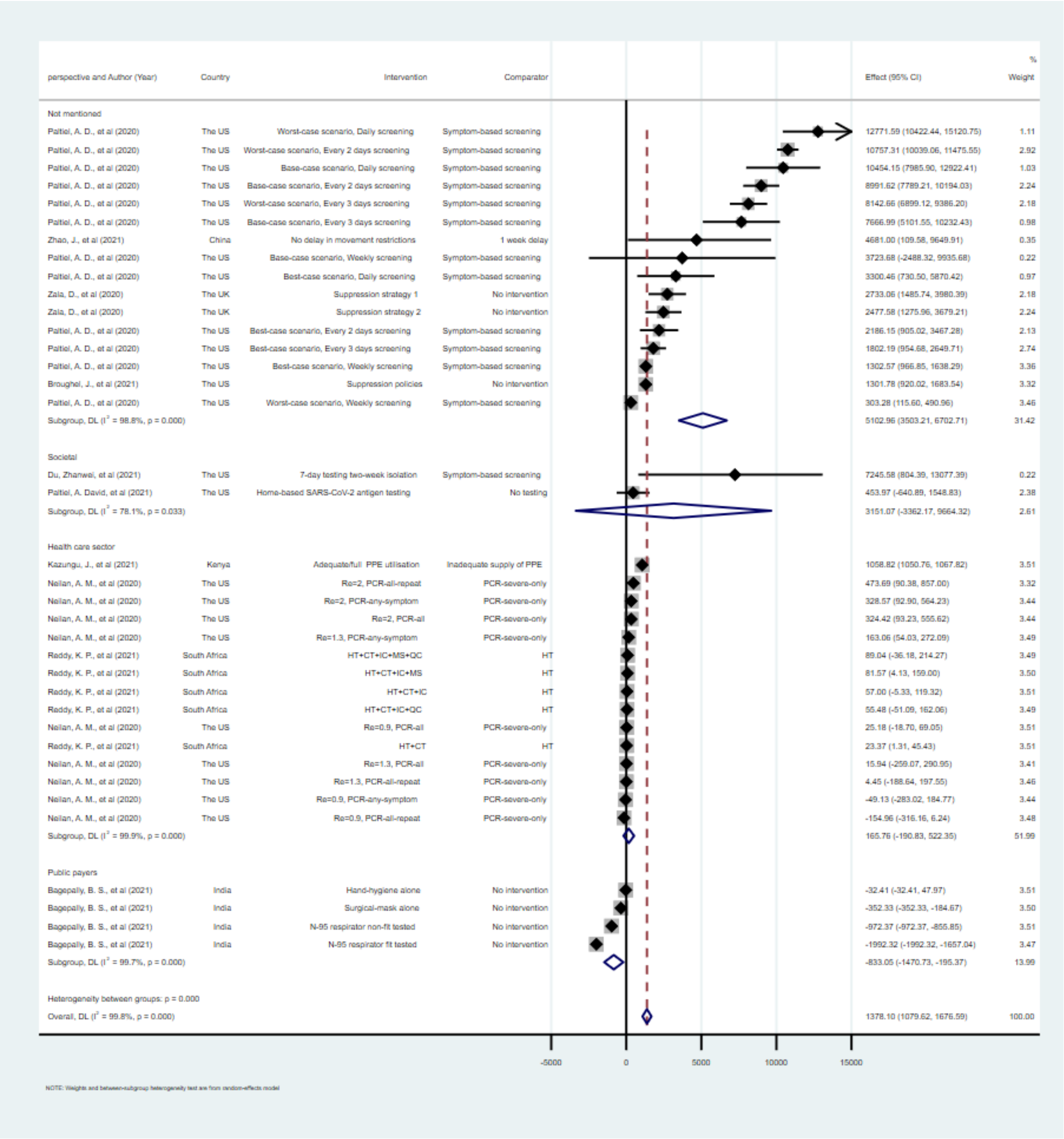

Figure S9 Subgroup analysis of the INB meta-analysis of NPIs of covid-19 in different perspective group.

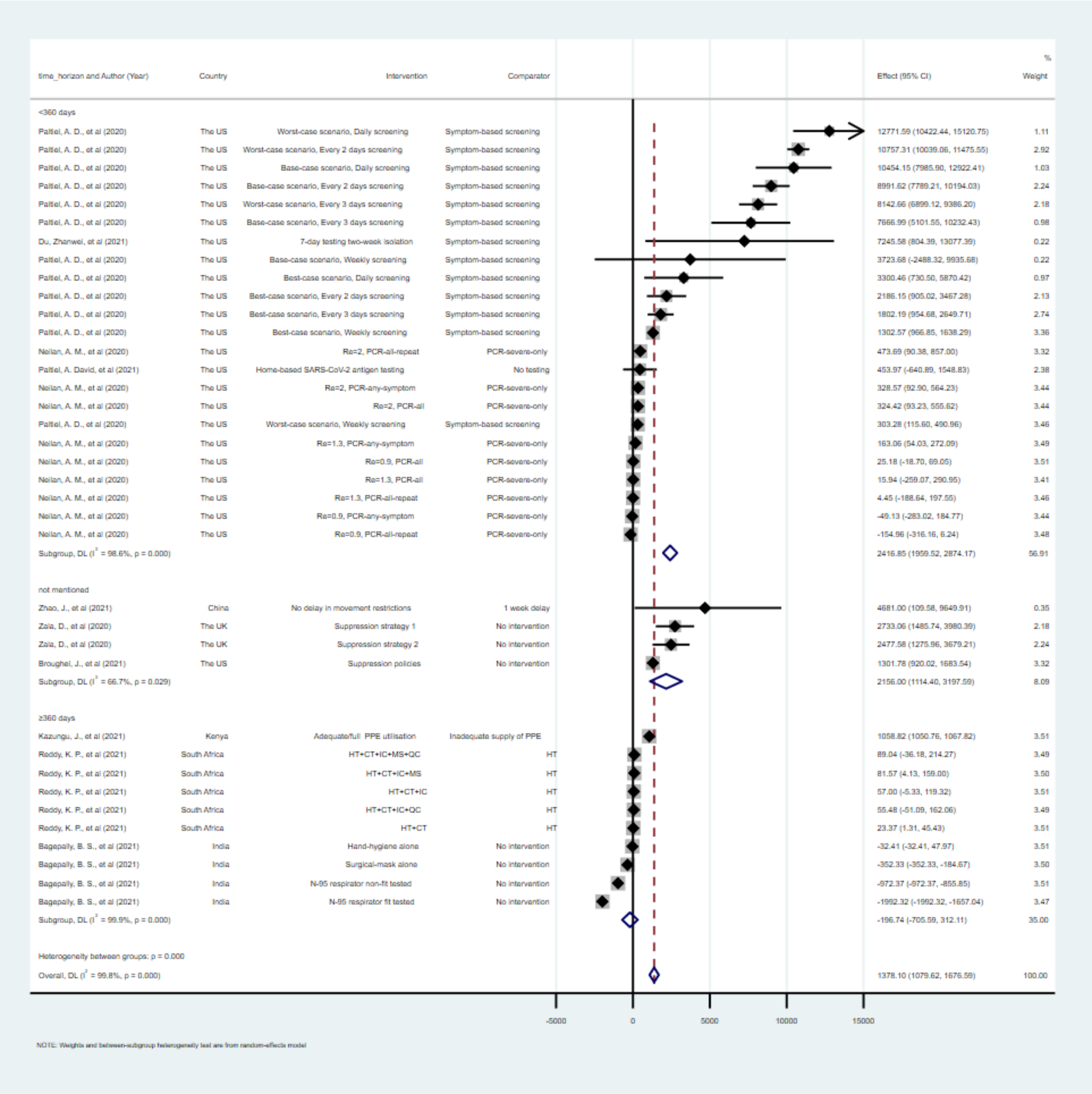

Figure S10 Subgroup analysis of the INB meta-analysis of NPIs of covid-19 in different time horizon group.

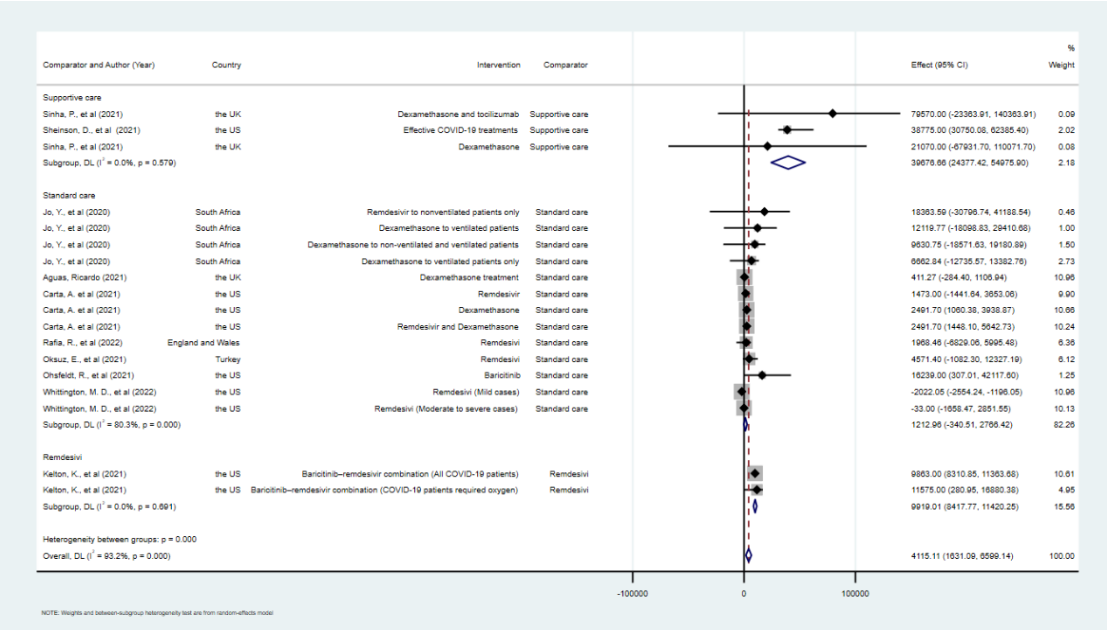

Figure S11 Subgroup analysis of the INB meta-analysis of covid-19 treatments in different comparator group.

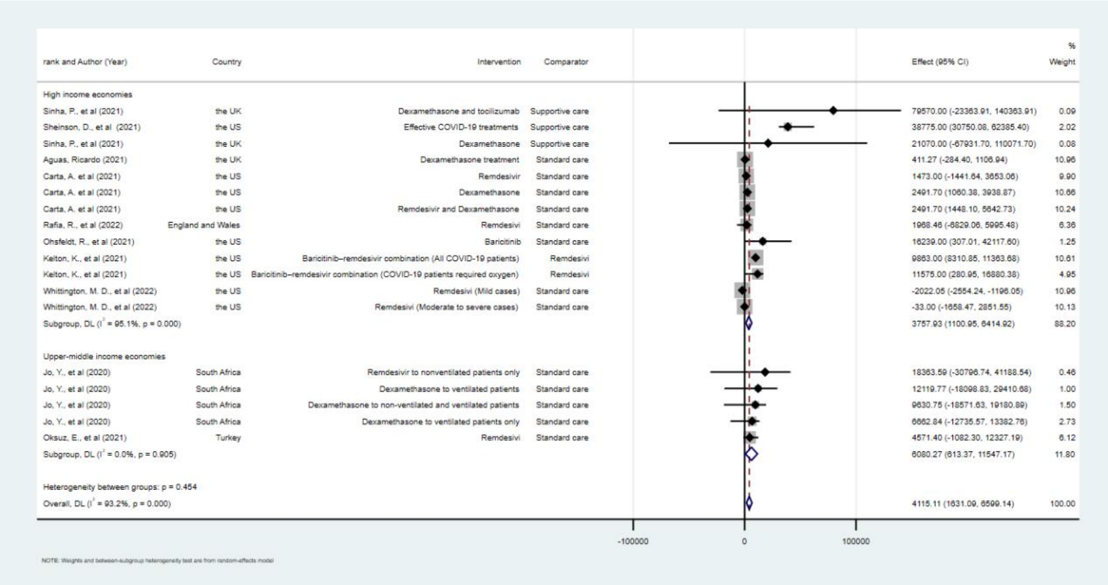

Figure S12 Subgroup analysis of the INB meta-analysis of covid-19 treatments in different income of economy group.

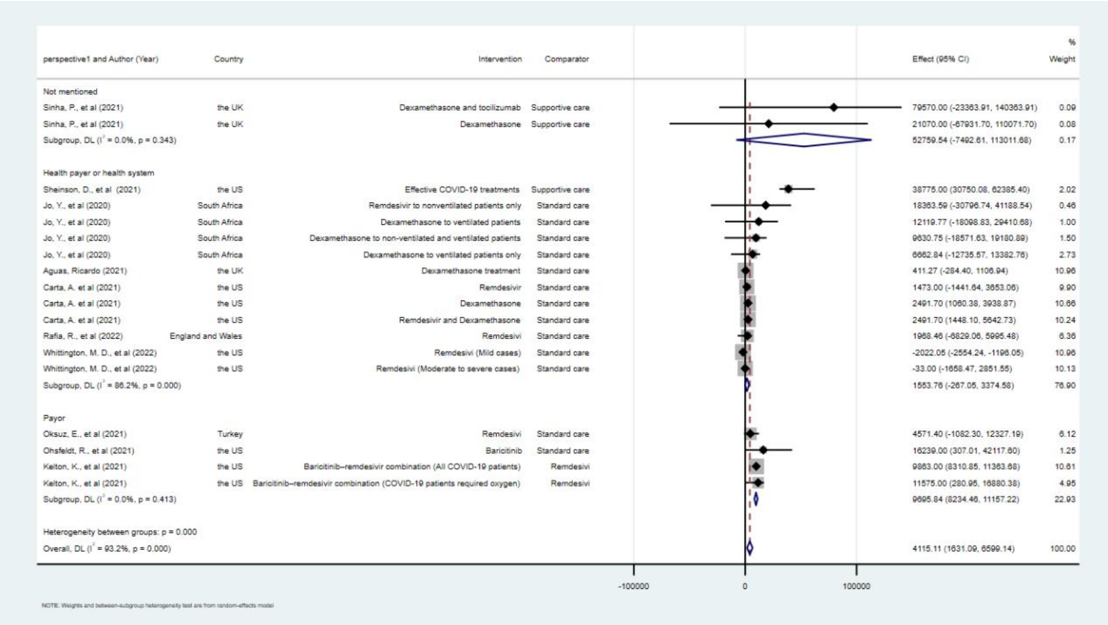

Figure S13 Subgroup analysis of the INB meta-analysis of covid-19 treatments in different perspective group.

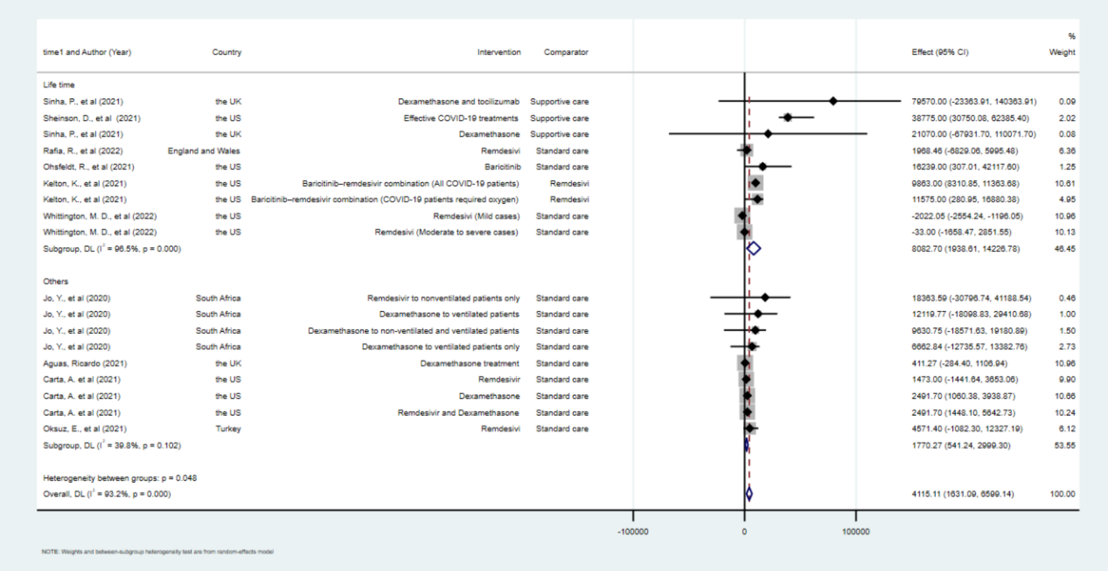

Figure S14 Subgroup analysis of the INB meta-analysis of covid-19 treatments in different time horizon group.

Table S3 Subgroup meta-analysis of vaccinations against COVID-19.

Subgroups	No. of studies	No. of interventions	INBp and 95% CI ($)	I^2 (%)	p (for I^2)
Type of comparators					
No vaccine	3	5	**294.97 (209.20, 380.75)**	97.7	<0.001
No booster	1	1	34.49 (-42.25, 111.23)	-	-
Risk-stratified vaccination	1	1	3018.07 (-7242.19, 13278.34)	-	-
Income of economies					
High income economies	4	6	**289.80 (202.89, 376.71)**	97.1	<0.001
Upper-middle income economies	1	1	**72.48 (6.58, 138.38)**	-	-
Perspectives					
Healthcare payer	3	5	**300.97 (104.40, 397.54)**	94.8	<0.001
Buyer	1	1	**248.08 (235.35, 260.81)**	-	-
Health care sector	1	1	**72.48 (6.58, 138.38)**	-	-
Time horizon					
≥360 days	2	2	**72.60 (6.70, 138.50)**	0.0	0.574
<360 days	3	5	**289.58 (202.49, 376.67)**	97.7	<0.001
Combined	5	7	**254.80 (169.84, 339.77)**	97.2	<0.001

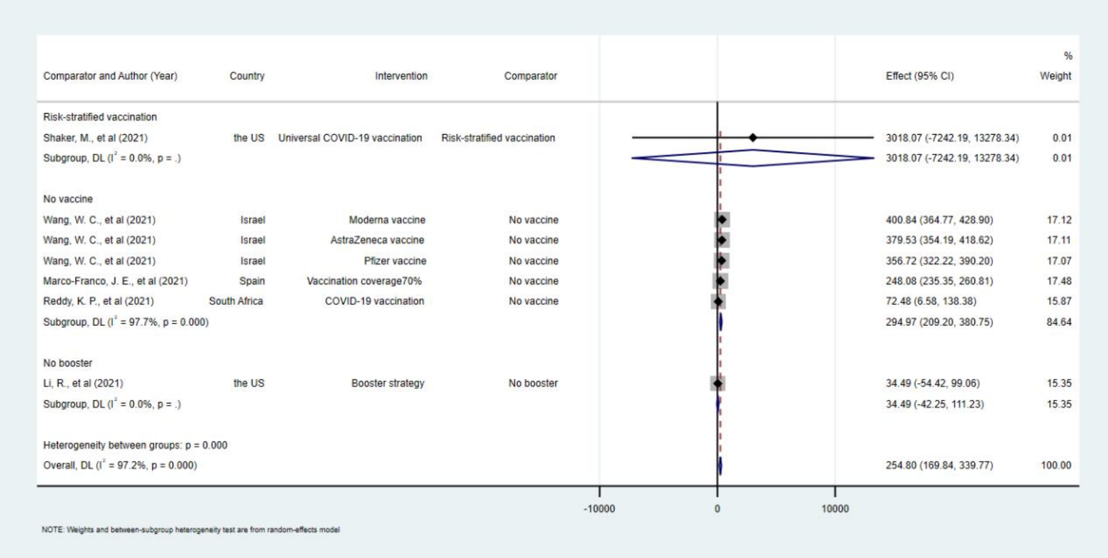

Figure S15 Subgroup analysis of the INB meta-analysis of covid-19 vaccinations in different comparator group.

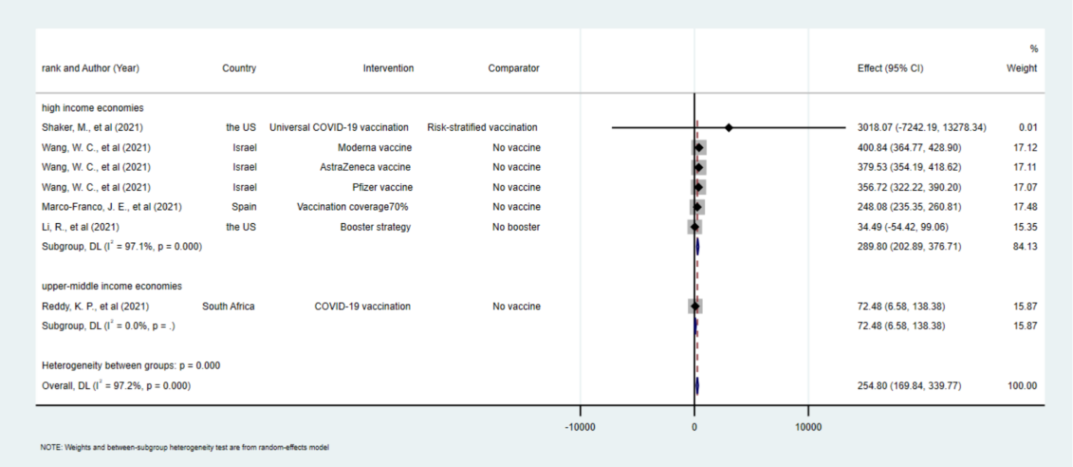

Figure S16 Subgroup analysis of the INB meta-analysis of covid-19 vaccinations in different income of economy groups.

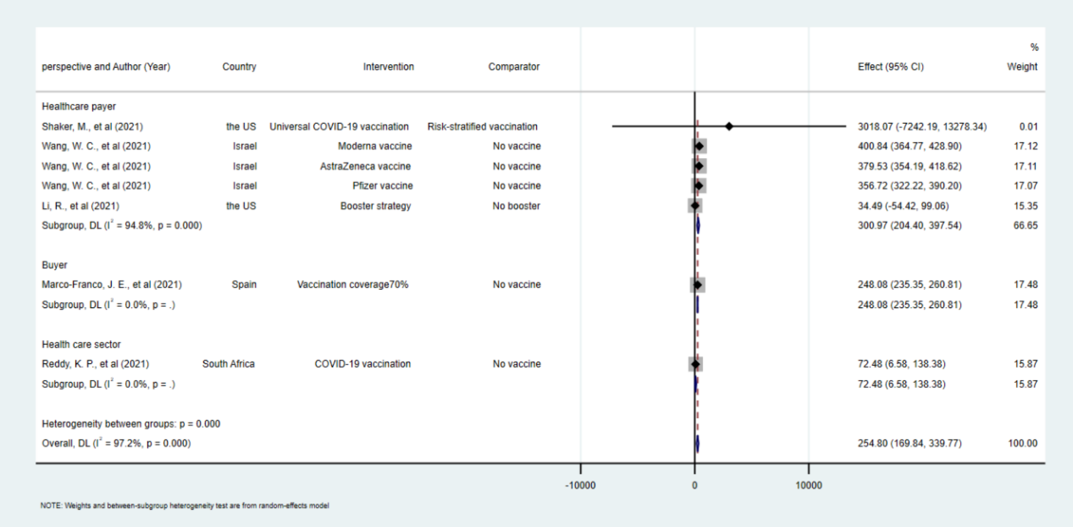

Figure S17 Subgroup analysis of the INB meta-analysis of covid-19 vaccinations in different perspective groups.

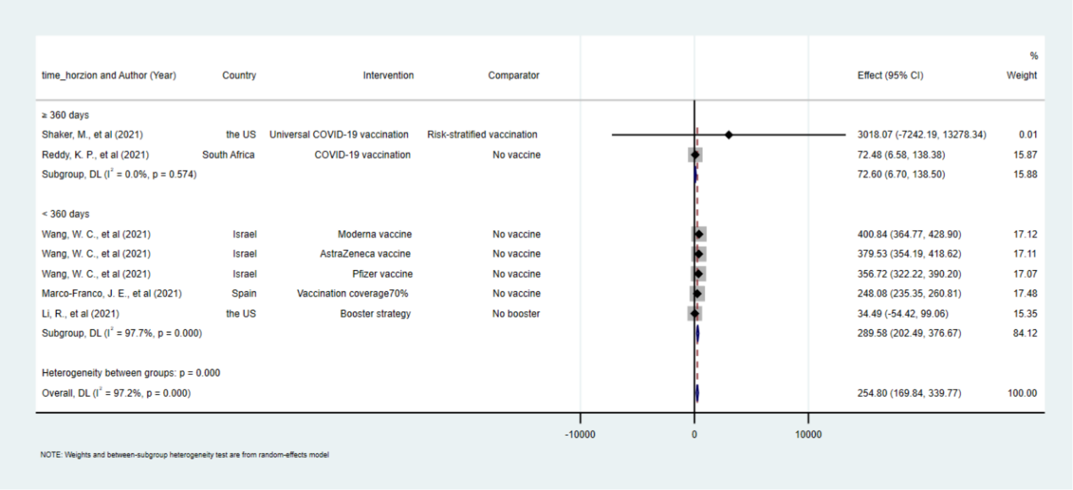

Figure S18 Subgroup analysis of the INB meta-analysis of covid-19 vaccinations in different time horizon groups.

Reference

1. Bagepally BS, Gurav YK, Anothaisintawee T, Youngkong S, Chaikledkaew U, Thakkinstian A. Cost Utility of Sodium-Glucose Cotransporter 2 Inhibitors in the Treatment of Metformin Monotherapy Failed Type 2 Diabetes Patients: A Systematic Review and Meta-Analysis. *Value in Health* 2019; **22**(12): 1458-69.
2. Chaiyakittisopon K, Pattanaprateep O, Ruenroengbun N, et al. Evaluation of the cost-utility of phosphate binders as a treatment option for hyperphosphatemia in chronic kidney disease patients: a systematic review and meta-analysis of the economic evaluations. *Eur J Health Econ* 2021; **22**(4): 571-84.
3. Bartsch SM, Wedlock PT, O'Shea KJ, et al. Lives and Costs Saved by Expanding and Expediting COVID-19 Vaccination. *J Infect Dis* 2021.
4. Bartsch SM, O'Shea KJ, Wedlock PT, et al. The Benefits of Vaccinating With the First Available COVID-19 Coronavirus Vaccine. *Am J Prev Med* 2021; **60**(5): 605-13.
5. Hagens A, İnkaya AÇ, Yildirak K, et al. Covid-19 vaccination scenarios: A cost-effectiveness analysis for turkey. *Vaccines* 2021; **9**(4).
6. Kirwin E, Rafferty E, Harback K, Round J, McCabe C. A Net Benefit Approach for the Optimal Allocation of a COVID-19 Vaccine. *Pharmacoeconomics* 2021.
7. Kohli M, Maschio M, Becker D, Weinstein MC. The potential public health and economic value of a hypothetical COVID-19 vaccine in the United States: Use of cost-effectiveness modeling to inform vaccination prioritization. *Vaccine* 2021; **39**(7): 1157-64.
8. Marco-Franco JE, Pita-Barros P, Gonzalez-de-Julian S, Sabat I, Vivas-Consuelo D. Simplified Mathematical Modelling of Uncertainty: Cost-Effectiveness of COVID-19 Vaccines in Spain. *Mathematics* 2021; **9**(5).
9. Padula WV, Malaviya S, Reid NM, et al. Economic value of vaccines to address the COVID-19 pandemic: a U.S. cost-effectiveness and budget impact analysis. *J Med Econ* 2021; **24**(1): 1060-9.
10. Reddy KP, Fitzmaurice KP, Scott JA, et al. Clinical outcomes and cost-effectiveness of COVID-19 vaccination in South Africa. *medRxiv* 2021.
11. Sandmann FG, Davies NG, Vassall A, Edmunds WJ, Jit M. The potential health and economic value of SARS-CoV-2 vaccination alongside physical distancing in the UK: a transmission model-based future scenario analysis and economic evaluation. *Lancet Infect Dis* 2021; **21**(7): 962-74.
12. Shaker M, Abrams EM, Greenhawt M. A Cost-Effectiveness Evaluation of Hospitalizations, Fatalities, and Economic Outcomes Associated with Universal Versus Anaphylaxis Risk-Stratified COVID-19 Vaccination Strategies. *J Allergy Clin Immunol Pract* 2021; **9**(7): 2658-68.e3.
13. Wang WC, Fann JC, Chang RE, et al. Economic evaluation for mass vaccination against COVID-19. *J Formos Med Assoc* 2021; **120 Suppl 1**: S95-s105.
14. López F, Català M, Prats C, et al. A Cost-Benefit Analysis of COVID-19 Vaccination in Catalonia. *Vaccines (Basel)* 2021; **10**(1).
15. Debrabant K, Grønbæk L, Kronborg C. The Cost-Effectiveness of a COVID-19 Vaccine in a Danish Context. *Clinical drug investigation* 2021; **41**(11): 975-88.
16. Pearson CAB, Bozzani F, Procter SR, et al. COVID-19 vaccination in Sindh Province, Pakistan: A modelling study of health impact and cost-effectiveness. *PLoS Med* 2021; **18**(10): e1003815.
17. Vaezi A, Meysamie A. COVID-19 Vaccines Cost-Effectiveness Analysis: A Scenario for Iran.

Vaccines (Basel) 2021; **10**(1).
18. Jiang Y, Cai D, Shi S. Economic evaluations of inactivated COVID-19 vaccines in six Western Pacific and South East Asian countries and regions: A modeling study. *Infect Dis Model* 2022; **7**(1): 109-21.
19. Kirson N, Swallow E, Lu J, et al. The societal economic value of COVID-19 vaccines in the United States. *J Med Econ* 2022; **25**(1): 119-28.
20. Du Z, Wang L, Pandey A, et al. Modeling comparative cost-effectiveness of SARS-CoV-2 vaccine dose fractionation in India. *Nat Med* 2022.
21. Liu Y, Sandmann FG, Barnard RC, et al. Optimising health and economic impacts of COVID-19 vaccine prioritisation strategies in the WHO European Region: a mathematical modelling study. *Lancet Reg Health Eur* 2022; **12**: 100267.
22. Bartsch SM, Wedlock PT, O'Shea KJ, et al. Lives and Costs Saved by Expanding and Expediting Coronavirus Disease 2019 Vaccination. *Journal of Infectious Diseases* 2021; **224**(6): 938-48.
23. Li R, Liu H, Fairley CK, et al. Cost-effectiveness analysis of BNT162b2 COVID-19 booster vaccination in the United States. 2021.
24. Aguas R, Mahdi A, Shretta R, et al. Potential health and economic impacts of dexamethasone treatment for patients with COVID-19. *Nature Communications* 2021; **12**(1).
25. Cleary SM, Wilkinson T, Tamandjou Tchuem CR, Docrat S, Solanki GC. Cost-effectiveness of intensive care for hospitalized COVID-19 patients: experience from South Africa. *BMC Health Serv Res* 2021; **21**(1): 82.
26. Gandjour A. How Many Intensive Care Beds are Justifiable for Hospital Pandemic Preparedness? A Cost-effectiveness Analysis for COVID-19 in Germany. *Appl Health Econ Health Policy* 2021; **19**(2): 181-90.
27. Jiang Y, Cai D, Chen D, Jiang S, Si L, Wu J. Economic evaluation of remdesivir for the treatment of severe COVID-19 patients in China under different scenarios. *Br J Clin Pharmacol* 2021.
28. Jo Y, Jamieson L, Edoka I, et al. Cost-effectiveness of remdesivir and dexamethasone for COVID-19 treatment in South Africa. *medRxiv* 2020.
29. Sheinson D, Dang J, Shah A, Meng Y, Elsea D, Kowal S. A Cost-Effectiveness Framework for COVID-19 Treatments for Hospitalized Patients in the United States. *Adv Ther* 2021; **38**(4): 1811-31.
30. Sinha P, Linas BP. Combination therapy with tocilizumab and dexamethasone cost-effectively reduces Coronavirus disease 2019 mortality. *Clin Infect Dis* 2021.
31. Carta A, Conversano C. Cost utility analysis of Remdesivir and Dexamethasone treatment for hospitalised COVID-19 patients - a hypothetical study. *BMC Health Serv Res* 2021; **21**(1): 986.
32. Rafia R, Martyn-St James M, Harnan S, Metry A, Hamilton J, Wailoo A. A Cost-Effectiveness Analysis of Remdesivir for the Treatment of Hospitalized Patients With COVID-19 in England and Wales. *Value Health* 2022.
33. Oksuz E, Malhan S, Gonen MS, et al. Cost-Effectiveness Analysis of Remdesivir Treatment in COVID-19 Patients Requiring Low-Flow Oxygen Therapy: Payer Perspective in Turkey. *Adv Ther* 2021; **38**(9): 4935-48.
34. Ohsfeldt R, Kelton K, Klein T, et al. Cost-Effectiveness of Baricitinib Compared With Standard of Care: A Modeling Study in Hospitalized Patients With COVID-19 in the United States. *Clin Ther* 2021; **43**(11): 1877-93.e4.

35. Jovanoski N, Kuznik A, Becker U, Hussein M, Briggs A. Cost-effectiveness of casirivimab/imdevimab in patients with COVID-19 in the ambulatory setting. *J Manag Care Spec Pharm* 2022: 1-11.
36. Kelton K, Klein T, Murphy D, et al. Cost-Effectiveness of Combination of Baricitinib and Remdesivir in Hospitalized Patients with COVID-19 in the United States: A Modelling Study. *Adv Ther* 2022; **39**(1): 562-82.
37. Whittington MD, Pearson SD, Rind DM, Campbell JD. The Cost-Effectiveness of Remdesivir for Hospitalized Patients With COVID-19. *Value Health* 2022.
38. Congly SE, Varughese RA, Brown CE, Clement FM, Saxinger L. Treatment of moderate to severe respiratory COVID-19: a cost-utility analysis. *Sci Rep* 2021; **11**(1): 17787.
39. Kairu A, Were V, Isaaka L, Agweyu A, Aketch S, Barasa E. Modelling the cost-effectiveness of essential and advanced critical care for COVID-19 patients in Kenya. *BMJ Glob Health* 2021; **6**(12).
40. Krylova O, Krasheninnikov A, Mamontova E, Tananakina G, Belyakova D. Pharmacoeconomic analysis of treatment regimens for coronavirus infection coronavirus disease-19. *Open Access Macedonian Journal of Medical Sciences* 2021; **9**: 1182-9.
41. Aldila D. Analyzing the impact of the media campaign and rapid testing for COVID-19 as an optimal control problem in East Java, Indonesia. *Chaos Solitons Fractals* 2020; **141**: 110364.
42. Asamoah JKK, Owusu MA, Jin Z, Oduro FT, Abidemi A, Gyasi EO. Global stability and cost-effectiveness analysis of COVID-19 considering the impact of the environment: using data from Ghana. *Chaos Solitons Fractals* 2020; **140**: 110103.
43. Bagepally BS, Haridoss M, Natarajan M, Jeyashree K, Ponnaiah M. Cost-effectiveness of surgical mask, N-95 respirator, hand-hygiene and surgical mask with hand hygiene in the prevention of COVID-19: Cost effectiveness analysis from Indian context. *Clin Epidemiol Glob Health* 2021; **10**: 100702.
44. Baggett TP, Scott JA, Le MH, et al. Clinical Outcomes, Costs, and Cost-effectiveness of Strategies for Adults Experiencing Sheltered Homelessness During the COVID-19 Pandemic. *JAMA Netw Open* 2020; **3**(12): e2028195.
45. Barnett-Howell Z, Watson OJ, Mobarak AM. The benefits and costs of social distancing in high- and low-income countries. *Transactions of the Royal Society of Tropical Medicine and Hygiene* 2021; **115**(7): 807-19.
46. Broughel J, Kotrous M. The benefits of coronavirus suppression: A cost-benefit analysis of the response to the first wave of COVID-19 in the United States. *PLoS One* 2021; **16**(6): e0252729.
47. Cook DC, Fraser RW, McKirdy SJ. A benefit-cost analysis of different response scenarios to COVID-19: A case study. *Health Sci Rep* 2021; **4**(2): e286.
48. Du Z, Pandey A, Bai Y, et al. Comparative cost-effectiveness of SARS-CoV-2 testing strategies in the USA: a modelling study. *Lancet Public Health* 2021; **6**(3): E184-E91.
49. Ebigbo A, Römmele C, Bartenschlager C, et al. Cost-effectiveness analysis of SARS-CoV-2 infection prevention strategies including pre-endoscopic virus testing and use of high risk personal protective equipment. *Endoscopy* 2021; **53**(2): 156-61.
50. Gandjour A. The Clinical and Economic Value of a Successful Shutdown During the SARS-CoV-2 Pandemic in Germany. *Q Rev Econ Finance* 2020.
51. Jiang Y, Cai D, Chen D, Jiang S. The cost-effectiveness of conducting three versus two reverse transcription-polymerase chain reaction tests for diagnosing and discharging people with COVID-19:

evidence from the epidemic in Wuhan, China. *BMJ Glob Health* 2020; **5**(7).
52. Khajji B, Kada D, Balatif O, Rachik M. A multi-region discrete time mathematical modeling of the dynamics of Covid-19 virus propagation using optimal control. *J Appl Math Comput* 2020: 1-27.
53. Kouidere A, Kada D, Balatif O, Rachik M, Naim M. Optimal control approach of a mathematical modeling with multiple delays of the negative impact of delays in applying preventive precautions against the spread of the COVID-19 pandemic with a case study of Brazil and cost-effectiveness. *Chaos Solitons Fractals* 2021; **142**: 110438.
54. Lim JT, Dickens BL, Cook AR, et al. The costs of an expanded screening criteria for COVID-19: A modelling study. *Int J Infect Dis* 2020; **100**: 490-6.
55. Lopez Segui F, Estrada Cuxart O, Mitja I Villar O, et al. A Cost-Benefit Analysis of the COVID-19 Asymptomatic Mass Testing Strategy in the North Metropolitan Area of Barcelona. *International journal of environmental research and public health* 2021; **18**(13).
56. Losina E, Leifer V, Millham L, et al. College Campuses and COVID-19 Mitigation: Clinical and Economic Value. *Ann Intern Med* 2021; **174**(4): 472-83.
57. Miles DK, Stedman M, Heald AH. "Stay at Home, Protect the National Health Service, Save Lives": A cost benefit analysis of the lockdown in the United Kingdom. *Int J Clin Pract* 2021; **75**(3): e13674.
58. Miles DK, Heald AH, Stedman M. How fast should social restrictions be eased in England as COVID-19 vaccinations are rolled out? *International Journal of Clinical Practice* 2021.
59. Neilan AM, Losina E, Bangs AC, et al. Clinical Impact, Costs, and Cost-Effectiveness of Expanded SARS-CoV-2 Testing in Massachusetts. *Clin Infect Dis* 2020.
60. Newbold SC, Finnoff D, Thunstrom L, Ashworth M, Shogren JF. Effects of Physical Distancing to Control COVID-19 on Public Health, the Economy, and the Environment. *Environmental & Resource Economics* 2020; **76**(4): 705-29.
61. Omame A, Sene N, Nometa I, et al. Analysis of COVID-19 and comorbidity co-infection model with optimal control. *Optim Control Appl Methods* 2021.
62. Paltiel AD, Zheng A, Walensky RP. Assessment of SARS-CoV-2 Screening Strategies to Permit the Safe Reopening of College Campuses in the United States. *JAMA Netw Open* 2020; **3**(7): e2016818.
63. Paltiel AD, Zheng A, Sax PE. Clinical and Economic Effects of Widespread Rapid Testing to Decrease SARS-CoV-2 Transmission. *Annals of Internal Medicine* 2021; **174**(6): 803-+.
64. Reddy KP, Shebl FM, Foote JHA, et al. Cost-effectiveness of public health strategies for COVID-19 epidemic control in South Africa: a microsimulation modelling study. *Lancet Glob Health* 2021; **9**(2): e120-e9.
65. Risko N, Werner K, Offorjebe OA, Vecino-Ortiz AI, Wallis LA, Razzak J. Cost-effectiveness and return on investment of protecting health workers in low- and middle-income countries during the COVID-19 pandemic. *PLoS One* 2020; **15**(10): e0240503.
66. Savitsky LM, Albright CM. Preventing COVID-19 Transmission on Labor and Delivery: A Decision Analysis. *Am J Perinatol* 2020; **37**(10): 1031-7.
67. Scherbina A. Assessing the Optimality of a COVID Lockdown in the United States. *Econ Disaster Clim Chang* 2021: 1-25.
68. Schonberger RB, Listokin YJ, Ayres I, Yaesoubi R, Shelley ZR. Cost Benefit Analysis of Limited Reopening Relative to a Herd Immunity Strategy or Shelter in Place for SARS-CoV-2 in the United States. *medRxiv* 2020.

69. Shlomai A, Leshno A, Sklan EH, Leshno M. Modeling Social Distancing Strategies to Prevent SARS-CoV-2 Spread in Israel: A Cost-Effectiveness Analysis. *Value in Health* 2021; **24**(5): 607-14.

70. Thunstrom L, Newbold SC, Finnoff D, Ashworth M, Shogren JF. The Benefits and Costs of Using Social Distancing to Flatten the Curve for COVID-19. *Journal of Benefit-Cost Analysis* 2020; **11**(2): 179-95.

71. Zala D, Mosweu I, Critchlow S, Romeo R, McCrone P. Costing the COVID-19 Pandemic: An Exploratory Economic Evaluation of Hypothetical Suppression Policy in the United Kingdom. *Value Health* 2020; **23**(11): 1432-7.

72. Zhao J, Jin H, Li X, et al. Disease Burden Attributable to the First Wave of COVID-19 in China and the Effect of Timing on the Cost-Effectiveness of Movement Restriction Policies. *Value Health* 2021; **24**(5): 615-24.

73. Gandjour A. Benefits, risks, and cost-effectiveness of COVID-19 self-tests from a consumer's perspective. *BMC Health Serv Res* 2022; **22**(1): 47.

74. Lally M. A cost-benefit analysis of COVID-19 lockdowns in Australia. *Monash bioethics review* 2022: 1-32.

75. Du Z, Wang L, Bai Y, et al. Cost-effective proactive testing strategies during COVID-19 mass vaccination: A modelling study. *Lancet Regional Health Americas* 2022; **8**: 100182.

76. Wang X, Cai Y, Zhang B, et al. Cost-effectiveness analysis on COVID-19 surveillance strategy of large-scale sports competition. *Infect Dis Poverty* 2022; **11**(1): 32.

77. de Assis TSM, Freire ML, Carvalho JP, Rabello A, Cota G. Cost-effectiveness of anti-SARS-CoV-2 antibody diagnostic tests in Brazil. *PLoS One* 2022; **17**(2): e0264159.

78. Zafari Z, Goldman L, Kovrizhkin K, Muennig PA. The cost-effectiveness of common strategies for the prevention of transmission of SARS-CoV-2 in universities. *PLoS One* 2021; **16**(9): e0257806.

79. Wang Q, Shi N, Huang J, et al. Cost-Effectiveness of Public Health Measures to Control COVID-19 in China: A Microsimulation Modeling Study. *Front Public Health* 2021; **9**: 726690.

80. Suwantika AA, Dhamanti I, Suharto Y, Purba FD, Abdulah R. The cost-effectiveness of social distancing measures for mitigating the COVID-19 pandemic in a highly-populated country: A case study in Indonesia. *Travel Med Infect Dis* 2022; **45**: 102245.

81. Maya S, Padda G, Close V, et al. Optimal strategies to screen health care workers for COVID-19 in the US: a cost-effectiveness analysis. *Cost Eff Resour Alloc* 2022; **20**(1): 2.

82. Asamoah JKK, Okyere E, Abidemi A, et al. Optimal control and comprehensive cost-effectiveness analysis for COVID-19. *Results Phys* 2022; **33**: 105177.

83. Shimul SN, Alradie-Mohamed A, Kabir R, Al-Mohaimeed A, Mahmud I. Effect of easing lockdown and restriction measures on COVID-19 epidemic projection: A case study of Saudi Arabia. *PLoS One* 2021; **16**(9): e0256958.

84. Kazungu J, Munge K, Werner K, Risko N, Vecino-Ortiz AI, Were V. Examining the cost-effectiveness of personal protective equipment for formal healthcare workers in Kenya during the COVID-19 pandemic. *BMC Health Serv Res* 2021; **21**(1): 992.

85. Bilinski A, Ciaranello A, Fitzpatrick MC, et al. SARS-CoV-2 testing strategies to contain school-associated transmission: model-based analysis of impact and cost of diagnostic testing, screening, and surveillance. *medRxiv* 2021.

86. Stevenson M, Metry A, Messenger M. Modelling of hypothetical SARS-CoV-2 point-of-care tests

on admission to hospital from A&E: Rapid cost-effectiveness analysis. *Health Technology Assessment* 2021; **25**(21): 1-68.
87. Thom H, Walker J, Vickerman P, Hollingworth W. Exploratory comparison of Healthcare costs and benefits of the UK's Covid-19 response with four European countries. *European Journal of Public Health* 2021; **31**(3): 628-+.
